# Reading an Artist’s Intention from the Composition (RAIC): eye movements and aesthetic experience in Japanese woodblock prints

**DOI:** 10.3389/fpsyg.2025.1644803

**Published:** 2025-11-12

**Authors:** Yuka Nojo, Antoni B. Chan

**Affiliations:** 1 Graduate School of Arts and Sciences, The University of Tokyo, Tokyo, Japan; 2 Department of Computer Science, City University of Hong Kong, Hong Kong SAR, China

**Keywords:** eye movements, aesthetic perception, gaze patterns, a variational Bayesian hierarchical extension of the EMHMM (VBHEM), Japanese woodblock prints (*Ukiyo-e*)

## Abstract

**Background:**

Understanding the cognitive mechanisms and decision-making processes involved in aesthetic judgement of visual art has become a growing focus in recent research. While eye movements have been strongly associated with impression evaluations, the underlying processes linking gaze behaviour and aesthetic experience remain underexplored. Recent discourse suggests that compositional strategies in artworks may guide viewers’ gaze and support narrative understanding.

**Objective:**

We hypothesised that the more closely a viewer’s gaze follows the artist’s intended compositional path, the better they comprehend the artwork’s intention and context, thereby enriching their aesthetic experience. This process is defined as RAIC (Reading an Artist’s Intention from the Composition).

**Methods:**

We collected 30-s eye-tracking data from 48 participants who viewed 12 Japanese woodblock landscape prints (*Ukiyo-e*). These artworks were selected from a preliminary study of 101 prints, based on the six highest and six lowest aesthetic ratings. Eye movements were segmented into 3-s intervals. Using the VBHEM algorithm, a variational Bayesian extension of the Eye Movement Hidden Markov Model (EMHMM), we evaluated the similarity between participants’ gaze sequences and expert-estimated scanpaths provided by specialists from the Japanese Painting Conservation and Restoration Laboratory of the Tokyo University of the Arts. Pupil size was analysed as an index of perceptual fluency.

**Results:**

Artworks with compositional structures aligned with expert scanpaths enabled viewers to better interpret the artist’s intention, promoting deeper aesthetic engagement. Additionally, high-rated artworks elicited greater perceptual fluency.

**Conclusion:**

These findings support the RAIC hypothesis, suggesting that guided visual exploration facilitates interpretation of artistic intention and contributes to a more meaningful aesthetic experience.

## 1 Introduction

Viewing a painting is an active process of visual information acquisition, and aesthetic judgements are formed from visual input within the first 6 s of viewing (Belfi et al., 2019; Schoeller and Perlovsky, 2016). Regarding the link between gaze and aesthetic appraisal, the area covered by eye movements reaches 56% of the painting by 7 s and increases little thereafter; thus, early fixations strongly influence subsequent appraisal (Locher et al., 2008). Sun et al. (2018) showed that specific landscape elements (e.g., landforms and architecture) and total fixation time relate to judgements of beauty, with longer viewing substantially increasing beauty ratings; similarly, Jankowski et al. (2020) reported a positive association between perceived beauty and dwell time, suggesting that viewers seek meaning by looking longer. Within the processing-fluency tradition, Leder et al. (2004) proposed that smoother perceptual processing increases preference; converging work indicates that aesthetic judgements are particularly sensitive to perceptual fluency (Reber et al., 2004; Vogel et al., 2020), which can be indexed by pupillary dynamics—greater dilation accompanying lower fluency and longer response times (Beatty and Lucero-Wagoner, 2000; Landgraf et al., 2010; Raisig et al., 2007, 2010; Verney et al., 2004). Complementing these mechanisms, compositional structure has long been proposed to guide attention and thereby shape viewing paths (Barhorst et al., 2023; Beelders and Bergh, 2020; Francuz et al., 2018; Sancarlo et al., 2020).

Evidence indicates that expertise modulates these processes. When viewing paintings—including abstract works—artists and other experts are less driven by low-level salience than novices, showing scanpaths that deviate from bottom-up saliency predictions and reflect top-down, knowledge-based exploration (Koide et al., 2015). Experts also adapt strategy to task demands and image type: relative to untrained viewers, they allocate greater attention to structural and compositional features and display distinct eye-movement patterns under free-viewing versus memory instructions (Vogt and Magnussen, 2007). Beyond gaze behaviour, expertise mitigates declines in aesthetic and emotional evaluations as abstraction increases; art-history experts maintain more stable ratings and exhibit different ocular and electrodermal responses across representational-to-abstract continua (Pihko et al., 2011). Concordantly, oculomotor correlates of expertise include differences in initial fixation allocation, scan efficiency, and dwell-time distribution during aesthetic appraisal (Francuz et al., 2018), and classic link formal art training to distinctive visual exploration and sensitivity to compositional balance (Nodine et al., 1993). Educational and interpretive interventions similarly shape viewing and comprehension. In museum contexts, age-appropriate, child-oriented descriptions measurably alter children’s eye movements and deepen engagement (Walker et al., 2024). In higher education, brief instruction modifies novices’ viewing strategies (Ishiguro et al., 2016). Gallery-level changes to display and interpretive layout also affect behaviour, with rearrangements increasing viewing time and label engagement (Reitstätter et al., 2020).

On the basis of this literature, we infer that (a) early fixations and perceptual fluency contribute substantially to aesthetic judgements, (b) expertise systematically reshapes gaze behaviour and affective–cognitive responses, and (c) educational/interpretive scaffolds can redirect eye movements and enhance understanding.

Thus, alongside expertise and interpretive scaffolds, composition itself functions as a device for directing viewers’ gaze. It is considered one of the elements that can influence the perceived “beauty” of a painting, as it contributes to visual order and balance (Fan et al., 2023). According to Hamm (1982), who drew a textbook on composition in landscape painting, a good landscape painting has four characteristics: (1) the viewer’s gaze never leaves the painting; (2) the painting has an introductory point for the gaze; (3) the gaze gradually moves from that introductory point through the near landscape to the back of the painting; and (4) the gaze returns to that introductory point and moves again after viewing the entire painting. Good composition can lead the viewer’s gaze through a through a goal-directed guidance mechanism, allowing them to understand the painting’s context (i.e., the artist’s intention) while allowing the gaze to move smoothly. In other words, composition is a device for manipulating the viewer’s gaze to understand the context of a painting. In fact, some studies (Garcia-Pelegrin et al., 2021) claim that art is a tool for storytelling that transcends time and space, and that a work of art has an intention or story that the artist is trying to convey through the work. Artists themselves have stated that “one should not paint with ambiguous intentions” (Hamm, 1982, p. 10) and that “an artist is an entertainer who spends a lifetime creating a story” (Murakami, 2018, p. 54).

The viewer is also likely to discern the artist’s intentions and background from the painting (Hartley and Allen, 2014). According to relevance theory (Sperber and Wilson, 1996; Wilson and Sperber, 2008), communication aims to achieve the greatest possible effect with the least possible cognitive effort for the recipient. Viewing a painting can be understood as a communicative act between the artwork and the viewer (Darda and Chatterjee, 2023). Extending this logic to the arts, artists seek to design works that maximise aesthetic impact while minimising the mental effort required for comprehension. Building on this premise, Crawford and Juricevic (2022) introduced the Corpus Analysis Relevance Theory (CART), which integrates relevance theory with corpus analysis. CART assumes that all paintings are figurative and interpretable through two complementary frameworks: a structural framework (the composition and visual elements such as size, shape, or position) and a contextual framework (the metaphorical or symbolic meanings assigned to those elements). By combining these frameworks, CART identifies four possible categories of interpretation: (1) Congruent-Literal, (2) Congruent-Metaphorical, (3) Incongruent-Structural Literal vs. Contextual Metaphorical, and (4) Incongruent-Structural Metaphorical vs. Contextual Literal. In their study, Crawford and Juricevic analysed 59 paintings and demonstrated that works in which structural cues aligned with contextual meanings were more readily and widely understood. Put differently, paintings that achieved a balance of lexical/structural clarity and contextual/metaphysical resonance elicited more consistent interpretations among viewers. This supports Starr’s (2023, p. 4) claim that “powerful aesthetic experiences occur when individuals have an unusually successful opportunity to make sense of the world around them.” In light of this framework, CART highlights how artworks function as communicative acts designed to optimise low cognitive effort and high communicative effect. This framework directly motivates our RAIC (Reading an Artist’s Intention from the Composition) hypothesis: that a viewer’s aesthetic experience depends on the efficiency with which gaze patterns enable them to access the artist’s intended meaning. While CART provides a theoretical framework for evaluating the communicative efficiency of artworks by analysing their structural and contextual congruence, RAIC extends this logic to the empirical level by examining whether viewers’ gaze patterns align with the artist’s compositional intentions, thereby shaping their aesthetic experience.

**Figure 1:**
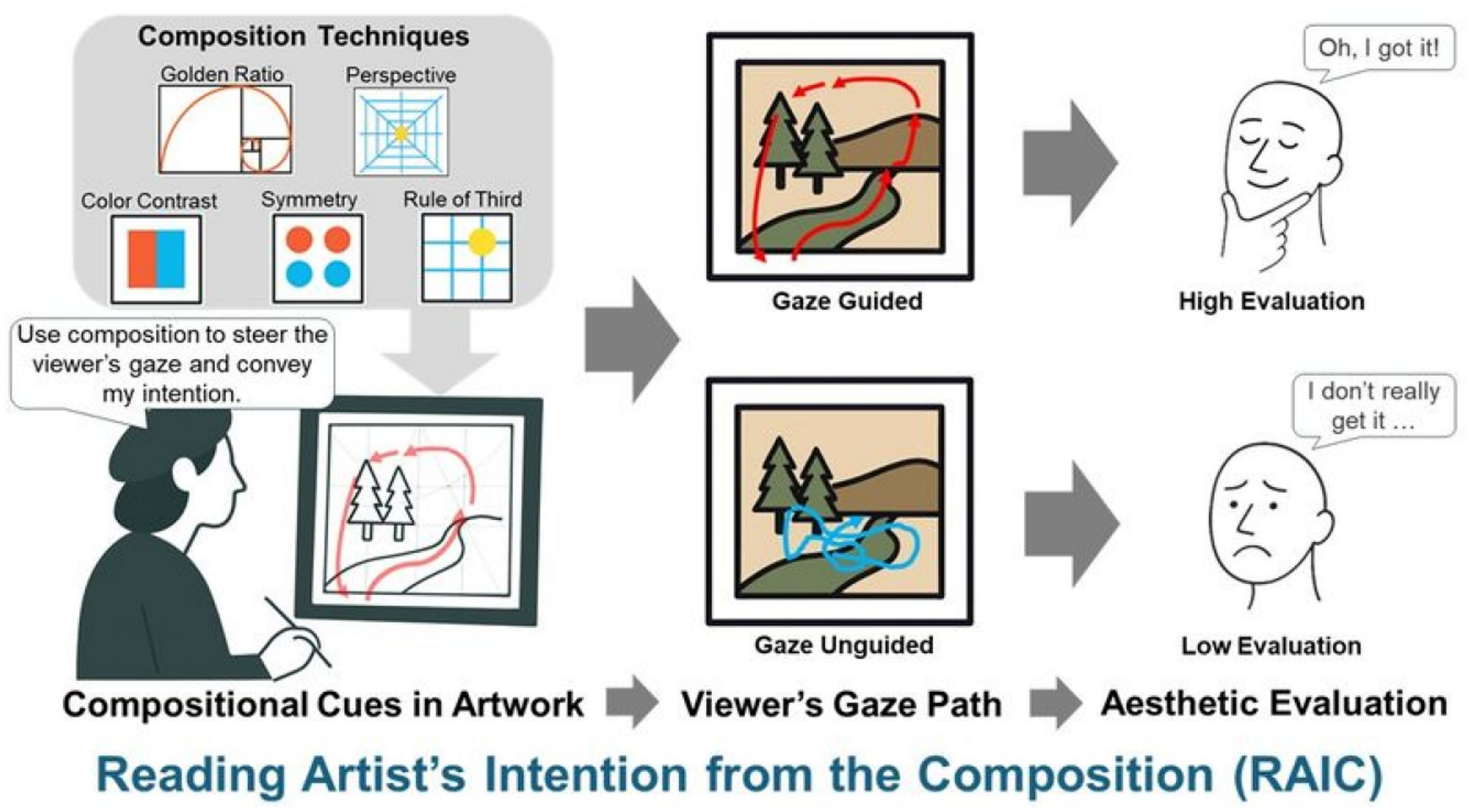
Conceptual framework of Reading an Artist’s Intention from the Composition (RAIC). Compositional techniques guide the viewer’s gaze, which can either align with the artist’s intention (gaze guided) or not (gaze unguided), resulting in differences in aesthetic evaluation.

As mentioned above, many previous studies have shown that the “beauty” of a painting is influenced by the quickness and clarity of perceptual processing. *Ukiyo-e* and related East Asian visual art have been underexplored in this regard. Here, we ask whether appropriate gaze patterns more effectively convey the artist’s intent and whether aesthetic experience improves when viewers engage with the work accordingly. To summarise this framework, Figure 1 presents a conceptual model of RAIC, highlighting its three core components: compositional cues, the viewer’s gaze path, and aesthetic evaluation. This model provides the theoretical basis for the present study and motivates the following hypothesis.

Considering Locher et al.’s (2008) findings, we hypothesized that “**The more the participant’s gaze moves as the expert expects it to while viewing the painting, the more successful the RAIC (Reading an Artist’s Intention from the Composition), the better the understanding of the world of the painting, and the better the aesthetic experience.**”

## 2 Materials and methods

In this study, *Ukiyo-e* was employed as a pictorial art form, which is a genre of painting that developed mainly in Japan between the 17th and 19th centuries and influenced 19th-century European art (especially French Impressionism) (cf. Bell, 2004; Harris, 2012). As experimental stimuli, Katsushika Hokusai’s “Thirty-six Views of Mount Fuji” (1831–1834) and Utagawa Hiroshige’s “Fifty-three Stages of the Tokaido” (c. 1834) were used (see Forrer, 2017; Nagata, 1995; Uhlenbeck and Jansen, 2008). Much of the research on aesthetic classification has focused on perspective-based images (using traditional Western painting techniques). However, *Ukiyo-e* differs from traditional Western techniques in several ways, such as not fully employing perspective and using a limited colour palette (Kadar and Effken, 2008). Therefore, it may be difficult to incorporate the “beauty” of *Ukiyo-e* into the image characteristics found in previous studies. These art-painting characteristics of *Ukiyo-e* have advantages for empirical study.

To verify the study hypothesis, we analysed the gaze data of participants while viewing these paintings. In particular, the participants were asked to view Katsushika Hokusai’s “Thirty-six Views of Mount Fuji” and Utagawa Hiroshige’s “Fifty-three Stages of the Tokaido.” Participants viewed 24 selected *Ukiyo-e* prints with eye-tracking and, after each image, completed five 10-point ratings, a Yes/No familiarity check, and a brief verbal comment; see Section 2.3 for full procedure. Gaze data were obtained using an eye-tracking device. The analysis focused on the relationship between the participants’ impression of the paintings and their eye movement.

Based on the experts’ gaze patterns, gaze data and impression ratings obtained in the experiment, three analyses were conducted to test the hypotheses. The first analysis compared the approximation rate between the experts’ gaze patterns (EGP) and the participants’ gaze patterns (PGP) via Eye Movement Hidden Markov Model (EMHMM) analysis (Hsiao et al., 2021; Chuk et al., 2014). The gaze data were subjected to variational Bayesian HMM modelling (Chuk et al., 2014) in 3 s increments to obtain the gaze transition probabilities for each participant, and then the variational Bayesian hierarchical expectation maximisation (VBHEM) (Lan et al., 2023) performed clustering to obtain common strategies, and the likelihood of the expert’s gaze pattern. Proximity was measured through the correlation between the likelihood and subjective impression ratings. The second analysis used pupil size and impression rating data to confirm the fluency for “beauty” processing. For each image, subjects were divided into high and low evaluation groups, and linear mixed model (LMM) was used to test whether there were significant differences in time series changes between groups in 3 s increments. The third analysis examined whether the artist’s intentions were being read, and the verbal impression evaluation was analysed with reference to the CART method. Referring to the CART framework outlined in the introduction, we defined RAIC (Reading an Artist’s Intention from the Composition) as successful when the verbal impression was metaphorical and contextually rich, and the gaze pattern closely aligned with that of the expert. This definition reflects the dual nature of *Ukiyo-e* as a realistic visual form that also allows room for metaphorical or symbolic interpretation by the viewer. As pointed out by Fenollosa (Yamaguchi, 1981), *Ukiyo-e* artists deliberately rejected idealised standards of beauty in favour of capturing everyday themes in a manner that allowed imaginative engagement. Based on this perspective, we adopted metaphorical expression as one of the key indicators of RAIC success. The success of RAIC was determined based on the following three criteria: (a) Whether the impression content matched the aesthetic rating as defined in Section 3.1 below (i.e., positive impressions for six highly rated works, and negative impressions for six low rated works); (b) Whether the impression was literal, describing structural features of the composition or depicted subjects; (c) Whether the impression was metaphorical, evoking imagination or suggesting interaction with the artist. RAIC was considered most successful when criterion (c) was strongly met and when gaze similarity (as in criterion a) was also high.

### 2.1 Participants

A total of 48 students (30 men and 18 women, *M_age_* = 22.2, *SD_age_* = 3.37) recruited from within and outside the University of Tokyo participated in the experiment. Owing to the large individual differences in gaze data, we assumed a medium effect size *r* = 0.30 (Cohen, 1988), transformed to *d* = 0.628. The data was divided into three groups (high, medium, and low) according to the “beauty” rating, and two of these groups (high and low) were subjected to a one-tailed test. We inferred that the PGP of the group with highly rated of “beauty” correlated more with the EGP than the group with lower rated. Under this assumption, a sample size of approximately 16 participants per group, for a total of 48 participants, is required for a power of 80%, *α* = 0.05. We assumed 48 participants because this number is consistent with previous research on gaze pattern analysis (Chuk et al., 2014). None of the participants were art specialists. The participants were paid a fixed amount as compensation after the experiment. The experiment was conducted in accordance with the ethical standards set forth by the ethics committee of the university to which the first author belongs.

### 2.2 Stimuli

A total of 101 candidate images were selected, including 46 from “Thirty-six Views of Mount Fuji” by Hokusai Katsushika, known for a “cartoon-like and illustrative style” (Ando, 1859, p. 2; Tsuji, 1972, pp. 78–79), and 55 from “The Fifty-three Stages of the Tokaido” by Hiroshige Utagawa, known for his “realistic style” (Ando, 1859; Tsuji, 1972) and as an *Ukiyo-e* artist active about the same time as Hokusai. All images were from a museum’s collection (image data source: The Sumida Hokusai Museum, Ota Memorial Museum of Art). “The Fifty-three Stages of the Tokaido” has decorative window-like frames at four corners; however, to standardise the shape of the experimental stimuli of the two painters, all frames were removed and stimuli without frames were prepared. The pilot experiment (see Supplementary material 1) was conducted with the aim of identifying the most and least appreciated landscape paintings by 46 of Katsushika Hokusai’s “Thirty-six Views of Mount Fuji” and 55 of Hiroshige’s “Fifty-three Stages of the Tokaido.” Participants viewed each painting for 30 s and rated various aspects such as composition and “beauty” on a 10-point scale; of the 101 paintings, six with particularly high impression ratings for “beauty” and “favourability” (mean = 7.85, SD = 0.70) and six with low impression ratings (mean = 4.34, SD = 0.62) were selected for this experiment employed as stimuli (mean = 6.17, SD = 2.17). Of the six highest rated paintings, four were by Katsushika Hokusai: “Under the Wave off Kanagawa” (commonly known as “The Great Wave” hereafter abbreviated as “Great Wave”), “Ejiri in Suruga Province” (hereafter abbreviated as “Ejiri”), “Under the Mannenbashi Bridge at Fukagawa” (hereafter abbreviated as “Mannenbashi”), and “Viewing Sunset over the Ryogokubashi Bridge from the Ommayagashi River Bank” (hereafter abbreviated as “Ryogokubashi”). The sixth “Shinagawa: Sunrise” (hereafter abbreviated as “Shinagawa”) and the seventh “Okazaki: Yahagi Bridge” (hereafter abbreviated as “Okazaki”) were by Utagawa Hiroshige. The six lowest rated works were “Sea Lane off Kazusa Province” (hereafter abbreviated as “Kazusa”) by Katsushika Hokusai; and “Futagawa: Sarugababa” (hereafter abbreviated as “Futagawa”), “Fujikawa: Scene at the Boundary Marker” (hereafter abbreviated as “Fujikawa”), “Okitsu: The Okitsu River” (hereafter abbreviated as “Okitsu”), “Fujieda: Changing Porters and Horses” (hereafter abbreviated as “Fujieda”), and “Akasaka: Inn with Serving Maids” (hereafter abbreviated as “Akasaka”) by Utagawa Hiroshige.

### 2.3 Procedure

**Figure 2:**
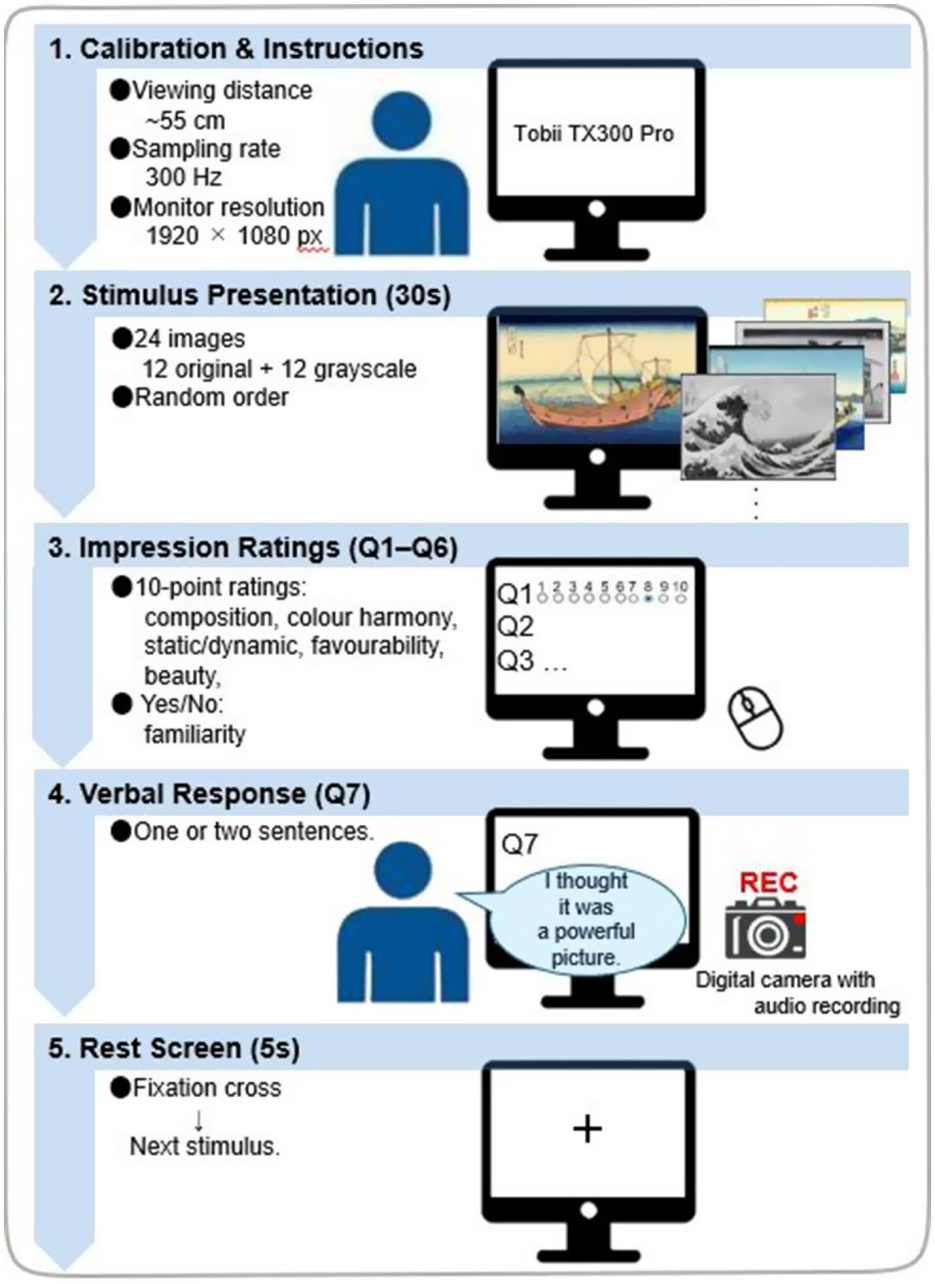
Experimental procedure. Participants sat ~55 cm from the monitor (Tobii TX300 Pro; 300 Hz; 1,920 × 1,080). Twenty-four images (12 original, 12 grayscale) were presented in randomised order for 30 s each while eye movements were recorded. After viewing, participants gave 10-point ratings (composition, colour harmony, static/dynamic, favourability, beauty) and answered in a Yes/No format whether they had seen the painting before, followed by a brief verbal impression (Q7, audio-recorded). Each trial ended with a 5 s fixation cross before the next stimulus.

The 24 images were individually presented on the PC display in a random order for 30 s; they consisted of 12 original and 12 grayscale images. The participants viewed the paintings on the PC display sequentially and rated their impressions on a 10-point scale (1 = most negative, 10 = most positive) for the following attributes in the order presented: the “quality of composition,” “integrity of colour harmony,” “static or dynamic,” “favourability,” and “beauty” (see Carbon, 2017). They then answered in a Yes/No format whether they had seen the painting before (see also Smith et al., 2017). All the aforementioned questions and response options were presented on the same display, and the participants responded using a mouse and without a time limit. The seventh question, “Please provide a verbal response in one or two sentences regarding your impression of the image,” was presented on the display. With the participants’ consent, their responses were recorded using an audio recording device. Upon completion of one *Ukiyo-e* rating, a rest screen was presented for 5 s. This screen featured a gazing point positioned centrally in a white background. This was followed by the next *Ukiyo-e*. The participants were instructed to maintain gaze at the gazing point during the presentation of the rest screen. The duration of viewing one *Ukiyo-e* was set to 30 s (cf. Carbon, 2017; Smith and Smith, 2001; Smith et al., 2017). The gaze of the participants while viewing the stimuli was recorded using the Tobii TX300 Pro eye marker recorder. Viewing and responding to the questions were combined into a single trial, with data collected for 24 images per participant. Figure 2 illustrates the experimental flow, including stimulus presentation, the rating sequence, verbal-response recording, the inter-trial fixation screen, and all timing parameters.

Regarding the transcription and coding of verbal responses, three independent coders—the first author and two external researchers, Xin Huang (Associate Professor, Department of Computer Science, Hong Kong Baptist University) and Bo Fang (Research Assistant Professor, Hong Kong Polytechnic University)—coded all verbatim transcripts using a pre-specified codebook (Literal: L1 Composition/Structure, L2 Colour—Descriptive, L3 Colour—Evaluative; Metaphorical: M1 Inference/Background meaning, M2 Personal evaluation/Associations). Inter-rater reliability varied across categories (Table 1). Substantial agreement was observed for ColourGood (*κ* = 0.664, 95% CI [0.584–0.739], full agreement = 0.901) and ColourBad (*κ* = 0.681, 95% CI [0.511–0.804], full agreement = 0.970). Moderate agreement was achieved for Difficult to understand (κ = 0.362, 95% CI [0.228–0.479], full agreement = 0.904) and Personal evaluation/Associations (*κ* = 0.420, 95% CI [0.375–0.466], full agreement = 0.784), while Inference/Background meaning showed fair-to-moderate agreement (*κ* = 0.321, 95% CI [0.284–0.355], full agreement = 0.737). Other categories demonstrated only fair agreement: Composition (*κ* = 0.183, 95% CI [0.124–0.246], full agreement = 0.791), Easy to understand (*κ* = 0.212, 95% CI [0.037–0.385], full agreement = 0.958), Explanation of colouring (*κ* = 0.234, 95% CI [0.155–0.312], full agreement = 0.758), and Impressions (*κ* = 0.211, 95% CI [0.158–0.264], full agreement = 0.409). Notably, full-agreement rates exceeded 0.75 in most categories, suggesting that κ values may be attenuated by category imbalance. At the multi-label level, the mean pairwise Jaccard similarity across responses was 0.494 (median = 0.333, SD = 0.314), and the mean of per-response medians was 0.357 (median = 0.250, SD = 0.405), indicating that coders’ label sets overlapped by nearly half on average. The full codebook and transcripts are provided in Supplementary material 2, and detailed inter-coder statistics are reported in Table 1.

**Table 1:** Inter-coder reliability for verbal-impression categories.

Category	*N*	Fleiss kappa	CI_L	CI_U	Full agreement multi
L1: Composition	575	0.1825	0.1242	0.2459	0.7913
L2: Difficult to understand	575	0.3619	0.2275	0.4787	0.9044
L3: Easy to understand	575	0.2116	0.0374	0.3852	0.9583
L4: Explanation of colouring	575	0.2337	0.1554	0.3121	0.7583
L5: Colour Good	575	0.6644	0.5837	0.7387	0.9009
L6: Colour Bad	575	0.6807	0.5110	0.8043	0.9704
L7: Impressions	575	0.2114	0.1576	0.2637	0.4087
M1: Inference/Background meaning	575	0.3205	0.2842	0.3552	0.7374
M2: Personal evaluation/Associations	575	0.4199	0.3754	0.4660	0.7844

Overall Jaccard (pairwise, per-response averaged)					
Mean of pairwiseJ means	Median of pairwiseJ means	SD of pairwiseJ means	Mean of pairwiseJ medians	Median of pairwiseJ medians	SD of pairwiseJ medians
0.4939	0.3333	0.3135	0.3568	0.2500	0.4051

### 2.4 Data analysis for gaze pattern

We analysed participants’ 30-s gaze data (Nojo et al., 2023). We extended the time series every 3 s, from 0 to 3, 0–6, and finally 0–30 s, and conducted a time series analysis of the gaze patterns at each time point. The relationship between the results and evaluation of impressions was investigated. As mentioned in the introduction, aesthetic judgements when viewing paintings are made within the first few seconds of viewing, and the distance of eye movement does not increase significantly after the initial viewing period. Based on this, we focus on the early phase of viewing, particularly the first few seconds, as an important time for aesthetic judgement. In this study, the objective was to elucidate the relationship between subjective impression evaluation and eye movement, and the analysis for the colour effects has been already reported (Nojo et al., 2023); thus, the grayscale was not focused on. For each image, data from those who exhibited a lack of fixation for a period exceeding two consecutive seconds were excluded (Table 1). Specifically, three from Great Wave; two from Okazaki and Fujikawa; and one each from Shinagawa, Ryogokubashi, Futagawa, and Okitsu were excluded.

For subjective impression ratings, data were normalised for each participant to account for the range of ratings observed between individuals. Furthermore, in order to identify any deviations in gaze patterns due to ratings, each image was divided into groups of high, medium, and low ratings; correlation coefficients with EGP were examined, particularly for the high and low ratings. To reflect the relative differences in the evaluation of each painting obtained in the pilot experiment, the normalised impression evaluation values of “beauty” and “favourability” were classified by k-means into three groups: high, medium, and low evaluation groups. The number of subjects in the three groups, per painting, is shown in Tables 2, 3.

**Table 2:** Number of participants per group by the impressions, per image; “Beauty.”

Title	High	Middle	Low	Excluded	Analysed	Total
Under the Wave off Kanagawa Commonly known as ‘Great Wave’	17	20	8	3	45	48
Shinagawa: Sunrise	21	17	9	1	47	48
Okazaki: Yahagi Bridge	23	15	8	2	46	48
Under the Mannenbashi Bridge at Fukagawa	18	20	10	0	48	48
Ejiri in Suruga Province	12	21	15	0	48	48
Viewing Sunset over the Ryogokubashi Bridge from the Ommayagashi River Bank	12	23	12	1	47	48
Futagawa: Sarugababa	10	23	14	1	47	48
Sea Lane off Kazusa Province	17	19	12	0	48	48
Okitsu: The Okitsu River	9	25	13	1	47	48
Fujikawa: Scene at the Boundary Marker	12	18	16	2	46	48
Fujieda: Changing Porters and Horses	10	20	18	0	48	48
Akasaka: Inn with Serving Maids	12	21	15	0	48	48

**Table 3:** Number of participants per group by the impressions, per image; “Favourability.”

Title	High	Middle	Low	Excluded	Analysed	Total
Under the Wave off Kanagawa Commonly known as ‘Great Wave’	10	16	19	3	45	48
Shinagawa: Sunrise	26	12	9	1	47	48
Okazaki: Yahagi Bridge	28	12	6	2	46	48
Under the Mannenbashi Bridge at Fukagawa	15	20	13	0	48	48
Ejiri in Suruga Province	19	22	7	0	48	48
Viewing Sunset over the Ryogokubashi Bridge from the Ommayagashi River Bank	21	17	9	1	47	48
Futagawa: Sarugababa	9	21	17	1	47	48
Sea Lane off Kazusa Province	18	14	16	0	48	48
Okitsu: The Okitsu River	10	22	15	1	47	48
Fujikawa: Scene at the Boundary Marker	18	19	9	2	46	48
Fujieda: Changing Porters and Horses	16	22	10	0	48	48
Akasaka: Inn with Serving Maids	15	13	20	0	48	48

### 2.5 Gaze patterns of *Ukiyo-e* experts

The gaze patterns of three art experts and one master’s student (a lecturer and a student, both currently enrolled in the Japanese Painting Conservation Laboratory of Tokyo University of the Arts, and two art preparatory school instructors who are graduates from the same laboratory of the university) were used as the Expert Gaze Pattern (EGP). As the experimental stimuli were from the Edo period, the specific gaze patterns assumed by the painters could not be ascertained. Conversely, it is reasonable to posit that experts in painting, particularly those specialising in Japanese-style painting and *Ukiyo-e*, possess a shared historical understanding of the compositional conventions associated with *Ukiyo-e* landscapes. The knowledge of compositional methods of painting is widely shared and generalised among painters in both Eastern and Western contexts. For example, during the Edo period in Japan, *Ukiyo-e* master Katsushika Hokusai published numerous textbooks, including “Ryakuga -Haya-Oshie” [One point lessons for rough drawing] (Katsushika, 1812), to instruct his students (Kobayashi, 1996). The compositional method of *Ukiyo-e* has been widely disseminated by Hokusai since the Edo period. Accordingly, we postulated that the EGPs of these experts would be analogous to those presumed by Katsushika Hokusai and Utagawa Hiroshige.

**Figure 3:**
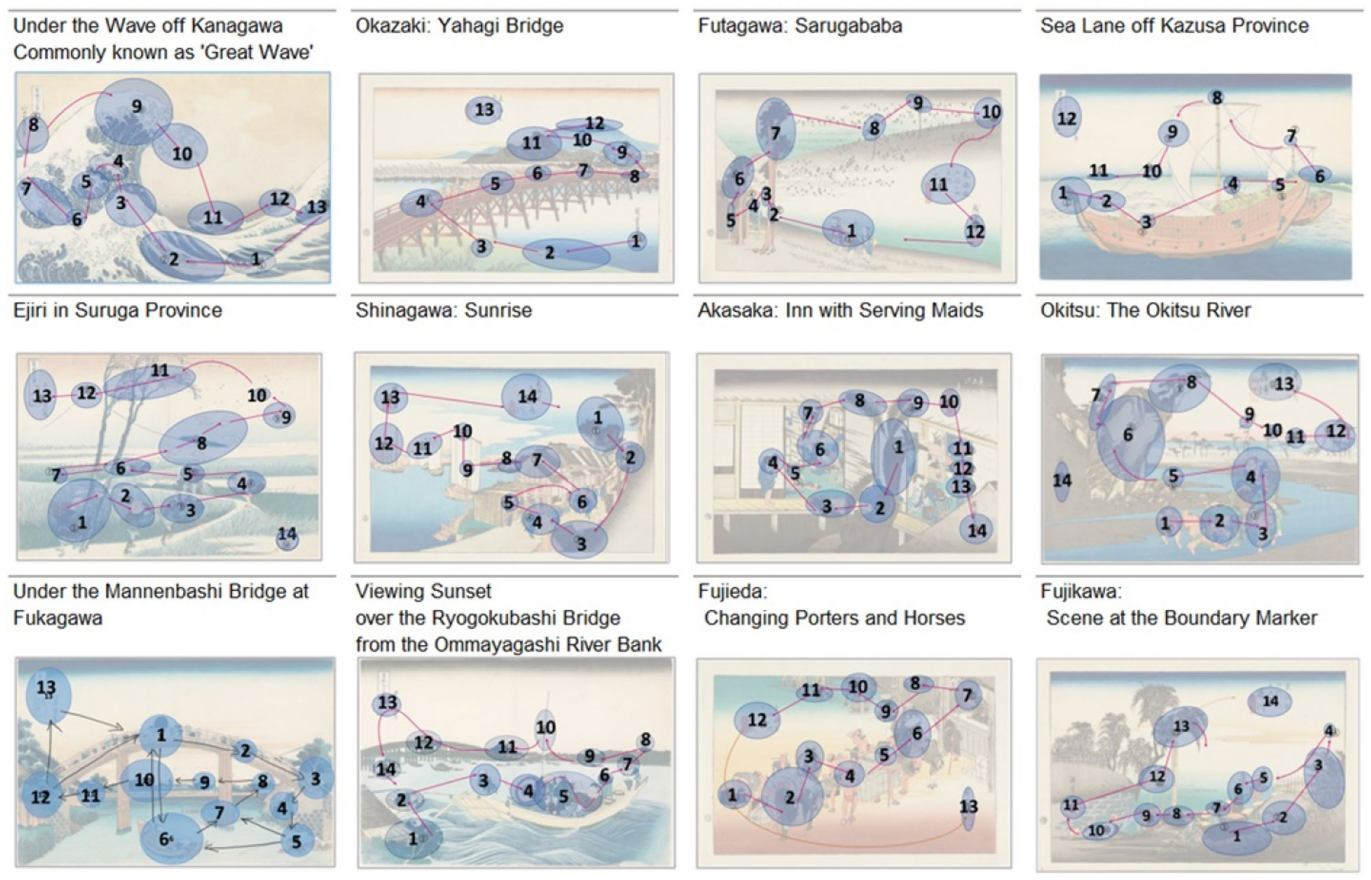
Gaze patterns of Ukiyo-e experts. The patterns for Great Wave, Ejiri, Ryogokubashi, Shinagawa, Okazaki, Kazusa, Akasaka, Okitsu, Fujikawa, Futagawa, and Fujieda were provided by a preparatory school instructor who graduated from the Japanese Painting Conservation and Restoration Laboratory of the Tokyo University of the Arts, whereas those for Mannenbashi were provided by a master’s student.

The experts manually annotated up to 14 regions of interest (ROIs) on each image and indicated their sequential order with arrows. Each expert’s marks were merged into a single binary mask; when adjacent ROIs occurred, one was recoloured red, and colour-separated regions were treated as distinct ROIs. Minimal preprocessing was applied, including small-blob removal, hole filling, and morphological opening. To avoid overgeneralised annotations, maps covering more than 50% of an image or exceeding 1.5 times the within-image median coverage were excluded. The representative ROI map for each image was defined as that of the annotator maximising the F1 score against a leave-one-out consensus mask (majority vote of the other experts). Ties were resolved by prioritising, in order: (i) maps with a higher ROI count, to avoid underspecification and preserve meaningful compositional subdivisions; (ii) greater IoU with the consensus; and (iii) coverage closer to the group median. Inter-expert reliability was quantified as Fleiss’ *κ* after downsampling masks to a 32 × 64 grid (cells marked present if any pixel was occupied). Across paintings, κ ranged from 0.10 to 0.36, the 3-of-4 consensus ratio was approximately 0.02–0.09, and the all-expert consensus ratio was approximately 0.002–0.006. These results were stable under sensitivity analyses using 24 × 48 and 16 × 32 grids and a 20% occupancy threshold (Table 4). In total, expert gaze patterns (EGPs) comprising 12–14 ROIs were obtained for the 12 paintings. The patterns for Great Wave, Ejiri, Ryogokubashi, Shinagawa, Okazaki, Kazusa, Akasaka, Okitsu, Fujikawa, and Fujieda were provided by a preparatory school instructor, whereas those for Mannenbashi were provided by a master’s student. As shown in Figure 3, Kazusa and Futagawa contained 12 ROIs, Great Wave, Mannenbashi, Okazaki, and Fujieda contained 13 ROIs; and the remaining works contained 14. All candidate EGPs are presented in Appendix 2, and the statistical results of inter-expert agreement are reported in Table 4.

**Table 4:** Statistical results of inter-expert agreement used to determine the representative ROI.

Artwork	nExperts	repExp	repComp Count	Mean IoU	Mean Dice	consensus 3of4	consensus all	repArea Frac	fleiss Kappa
Under the Wave off Kanagawa Commonly known as ‘Great Wave’	4	**2**	**13**	0.1828	0.2824	0.0917	0.0025	0.2834	0.1994
Shinagawa: Sunrise	4	**2**	**14**	0.1697	0.2663	0.0634	0.0036	0.1864	0.2523
Okazaki: Yahagi Bridge	4	**2**	**13**	0.1399	0.2310	0.0408	0.0019	0.1340	0.1931
Under the Mannenbashi Bridge at Fukagawa	4	**4**	**13**	0.1682	0.2625	0.0646	0.0026	0.2261	0.2162
Ejiri in Suruga Province	4	**2**	**12**	0.1127	0.1779	0.0227	0.0017	0.0764	0.0965
Viewing Sunset over the Ryogokubashi Bridge from the Ommayagashi River Bank	4	**2**	**14**	0.1941	0.2980	0.0702	0.0053	0.1601	0.2784
Futagawa: Sarugababa	4	**2**	**12**	0.2271	0.3332	0.0548	0.0055	0.1311	0.3565
Sea Lane off Kazusa Province	4	**2**	**11**	0.1317	0.2180	0.0414	0.0008	0.0762	0.1707
Okitsu: The Okitsu River	4	**2**	**14**	0.2083	0.3097	0.0916	0.0053	0.2524	0.2493
Fujikawa: Scene at the Boundary Marker	4	**2**	**13**	0.1624	0.2569	0.0617	0.0020	0.1747	0.2165
Fujieda: Changing Porters and Horses	4	**2**	**13**	0.1943	0.2983	0.0788	0.0059	0.1714	0.2637
Akasaka: Inn with Serving Maids	4	**2**	**14**	0.1562	0.2472	0.0426	0.0012	0.1644	0.2294

EMHMM analysis (Chuk et al., 2014; Hsiao et al., 2021) was used to compare the approximation rates of eye gaze patterns between experts and participants. The model uses a Bayesian framework and clustering of individual models to obtain common patterns (strategies), allowing the analysis to reflect individual differences. In addition, as the similarity between patterns is quantified by log-likelihood, it is possible to analyse the relationship between gaze patterns and other measures such as impression ratings; thus, the analysis tool was considered the most appropriate for our analysis. In addition, it is necessary to always estimate the transition probability of all ROIs, instead of limiting the direction of travel in which the gaze proceeds to one. This can also be handled by EMHMM; the fact that the position and order of ROIs can be fixed in advance was an important reason EMHMM was selected as the analysis method. In this study, the EGP was used as the fixed ROI and the transition probabilities of the participant’s gaze were extracted. First, all ROIs in the EGP were masked, and the centre coordinates, lengths of major and minor axes, and slope of the ROIs were calculated using the regionprops function in MATLAB R2023a. Based on these four values, the order and position of the ROIs for each painting were pre-specified, and the PGP was extracted using HMM with the fixations. However, fine angle adjustment (>90 degrees) was not performed to reduce the computational load of the matrix. All images were processed using the same parameters. Gaze data were processed in 3 s increments using the VBHEM (Chuk et al., 2014) algorithm to estimate the HMM for each individual and obtain transition probabilities from PGP to EGP for each participant. Then, VBHEM (Lan et al., 2023) was used to perform clustering of individuals into two groups, based on strategy. The results yielded a representative transition probability for a single image, and two types of transition probabilities were further classified automatically. The latter captures the characteristic behaviour of participants and allows for a more representative and detailed comparison of the behavioural patterns within the group. Therefore, we used these two types of results and compared their similarity to the eye movements of each participant. The correlation between the obtained transition probabilities and subjective impression ratings was analysed: the log-likelihood of the EGP and each participant’s transition probability (hereinafter referred to as VH-LL) and the correlation coefficient of the impression rating of “beauty” (hereinafter referred to as CVB) were calculated for each image. Notably, as the ROI was fixed this time, no differences in hidden states are generated, and comparisons based on transition probabilities constitute the primary focus.

### 2.6 EMHMM-based modelling and VH-LL/CVB metrics (gaze–rating link)

**Figure 4:**
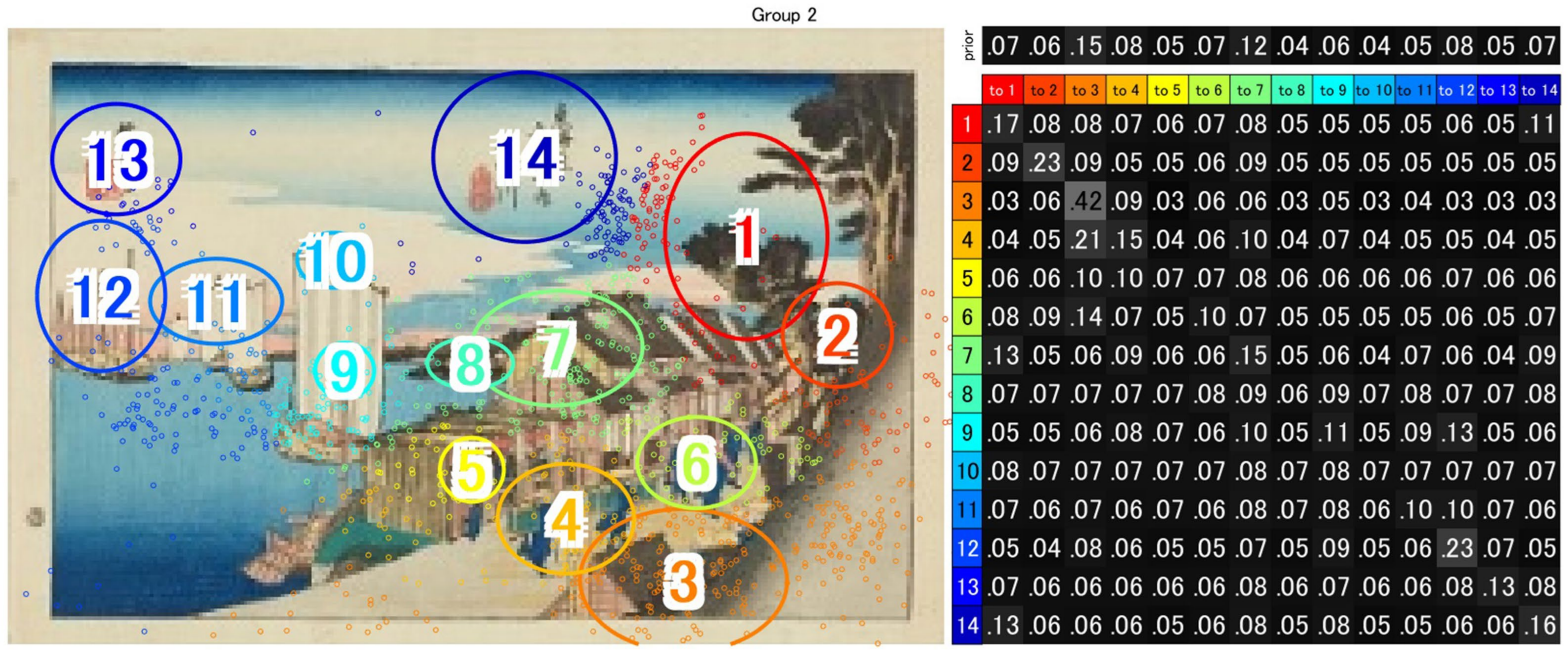
The transition probabilities for Shinagawa over the first 12 s. ROIs are arranged according to the sequence, size, and position defined by the artist’s gaze pattern (EGP). Each cell represents the probability of transitioning from one ROI (row) to another (column). Cells with higher probabilities indicate that fixations were repeatedly made within a limited subset of ROIs, suggesting that gaze transitions were not uniform but concentrated on specific regions.

We estimated gaze-transition models using EMHMM and obtained three types: (i) a global representative HMM, (ii) cluster-based representative HMMs—(a) a focused strategy, reflecting repeated fixations on a small subset of ROIs, and (b) an explorative strategy, reflecting broader scanning across multiple ROIs—and (iii) individual HMMs for each participant. For hypothesis testing, we adopted the two cluster-based representative models (a. focused and b. explorative), as they capture strategy-level structures while accommodating inter-individual variability. As an illustration, Figure 4 presents the transition probabilities for Shinagawa over the first 12 s.

The 12 images were classified into high- and low-rated groups based on the sign of the normalised “beauty” scores. At each time point, group comparisons (high-rated vs. low-rated) were conducted using F-tests to examine homogeneity of variance; when satisfied, t-tests were applied, otherwise Wilcoxon tests. Furthermore, to quantify the relationship between gaze and evaluation, VH-LL and CVB were computed at 3-s intervals (3, 6, 9, 15, 30 s) using the normalised impression ratings.

**Figure 5:**
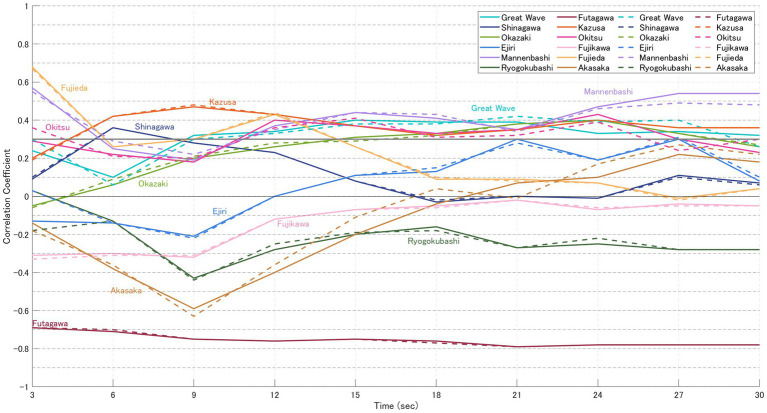
CVB for “beauty” (participants in the high-evaluation group). Solid lines represent the focused model **(a)**, and dashed lines represent the explorative model **(b)**. Each point represents the mean CVB of participants who rated the 12 paintings higher for “beauty” (see Table 2), calculated at 3, 6, 9, 15, and 30 s. At 3 s, nine of the 12 paintings showed a pattern consistent with the hypothesis, and significant differences were observed in several works including Ryogokubashi, Futagawa, Okitsu, and Akasaka at 6 s.

**Figure 6:**
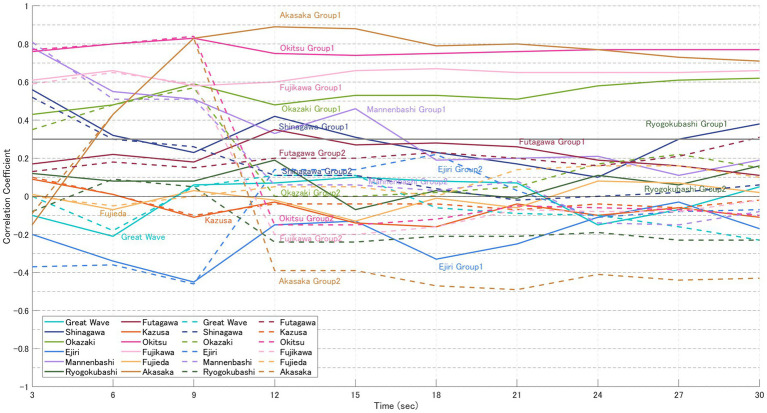
CVB for “beauty” (participants in the low-evaluation group). Solid lines represent the focused model **(a)**, and dashed lines represent the explorative model **(b)**. Each point represents the mean CVB of participants who rated the 12 paintings lower for “beauty” (see Table 2), calculated at 3, 6, 9, 15, and 30 s. In some prints, such as Okitsu, Fujikawa, and Akasaka, the low-evaluation group exhibited equal or greater alignment with expert gaze patterns, suggesting that compositionally guided viewing may also intensify negative impressions.

**Figure 7:**
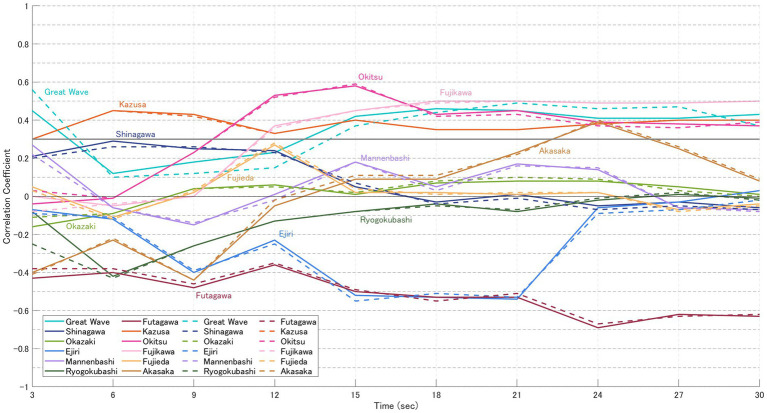
CVB for “favourability” (participants in the high-evaluation group). Solid lines represent the focused model **(a)**, and dashed lines represent the explorative model **(b)**. Each point represents the mean CVB of participants who rated the 12 paintings higher for “favourability” (see Table 3), calculated at 3, 6, 9, 15, and 30 s.

**Figure 8:**
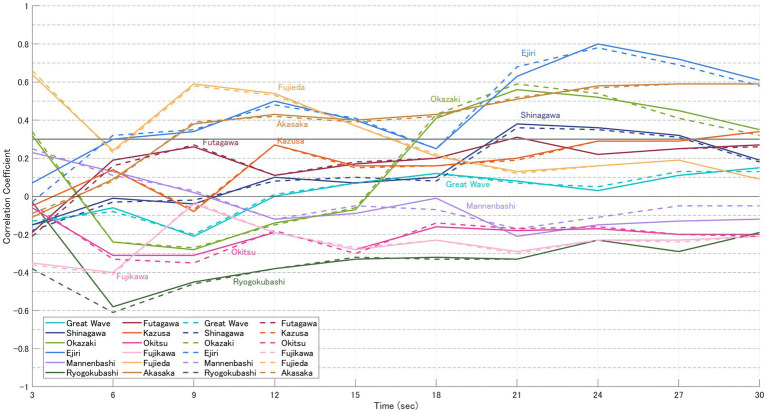
CVB for “favourability” (participants in the low-evaluation group). Solid lines represent the focused model **(a)**, and dashed lines represent the explorative model **(b)**. Each point represents the mean CVB of participants who rated the 12 paintings lower for “favourability” (see Table 3), calculated at 3, 6, 9, 15, and 30 s. In contrast, the low-evaluation group (Akasaka, Okazaki, Ryogokubashi, Kazusa, Okitsu, Fujikawa, and Fujieda) tended to show stronger CVB under the explorative model, indicating weaker correspondence with focused expert strategies.

For each image, the likelihood based on gaze-transition probabilities (VH-LL) and the CVB derived from normalised impression ratings of “beauty” and “favorability” were calculated. The results are presented in Figures 5–8. In these graphs, solid lines represent the focused model (a), while dashed lines represent the explorative model (b). The hypothesis of this study was that the closer participants’ gaze patterns were to the expert-defined patterns, the more strongly the artist’s intention would be conveyed, thereby deepening the aesthetic experience. In other words, paintings with a positive message would be rated higher, while those with a negative message would be rated lower. Specifically, the analysis was based on the following assumptions:

Case for “beauty” (Table 5)

- 6 highly-rated (Great Wave, Shinagawa, Okazaki, Mannenbashi, Ejiri, Ryogokubashi): CVB in Figure 5 > CVB in Figure 6 → Consistent with the hypothesis.
- 6 low-rated (Futagawa, Kazusa, Okitsu, Fujikawa, Fujieda, Akasaka): CVB in Figure 5 < CVB in Figure 6 → Consistent with the hypothesis.

**Table 5:** Normalised “Beauty” and correlation of VH-LL and “Beauty.”

	Title	Normalised ‘Beauty’					*		Correlation of VH-LL and ‘Beauty’																	
		Rank	Overall Average				3		6		9		12		15		18		21		24		27		30	
				High	Middle	Low	G1	G2	G1	G2	G1	G2	G1	G2	G1	G2	G1	G2	G1	G2	G1	G2	G1	G2	G1	G2
Highly rated	Under the Wave off Kanagawa Commonly known as ‘Great Wave’	**1**	**0.74**	1.66	0.85	−0.29	D	D			✓		✓	✓	✓	✓	✓	✓	✓	✓	✓	✓	✓	✓	✓	✓
	Shinagawa: Sunrise	**2**	**0.21**	1.13	0.27	−0.77											X	X	X	X	X	X	X	X	X	X
	Ejiri in Suruga Province	**3**	**0.21**	1.11	0.28	−0.77											✓		✓		✓		✓		✓	
	Under the Mannenbashi Bridge at Fukagawa	**4**	**0.16**	1.23	0.14	−0.89	D	D																		
	Viewing Sunset over the Ryogokubashi Bridge from the Ommayagashi River Bank	**5**	**0.08**	1.12	0.19	−1.07	D	D	✓				X		X		X		X		X		X		X	
	Okazaki: Yahagi Bridge	**6**	**0.07**	1.13	0.10	−1.01	D				X	X	X	X	X	X	X	X	X	X	X	X	X	X	X	X
Low rated	Futagawa: Sarugababa	**7**	**−0.06**	1.00	−0.05	−1.12	D	D	✓	✓	✓	✓	✓	✓	✓	✓	✓	✓	✓	✓	✓	✓	✓	✓	✓	✓
	Fujieda: Changing Porters and Horses	**8**	**−0.32**	0.82	−0.37	−1.41	D	D			X	X	X	X	X	X	X	X	X	X	X	X	X	X	X	X
	Sea Lane off Kazusa Province	**9**	**−0.36**	0.88	−0.34	−1.62	D	D			X	X	X	X	X	X	X	X	X	X	X	X	X	X	X	X
	Okitsu: The Okitsu River	**10**	**−0.36**	0.61	−0.42	−1.28	D	D	✓	✓	✓	✓	✓	✓	✓	✓	✓	✓	✓	✓	✓	✓	✓	✓	✓	✓
	Akasaka: Inn with Serving Maids	**11**	**−0.54**	0.37	−0.51	−1.47									✓	✓	✓	✓	✓	✓	✓	✓	✓	✓	✓	✓
	Fujikawa: Scene at the Boundary Marker	**12**	**−0.50**	0.58	−0.49	−1.61	D	D	✓	✓	✓	✓	✓	✓	✓	✓	✓	✓	✓	✓	✓	✓	✓	✓	✓	✓

Highly rated.

✓ = As hypothesized, the high ratings for the painting show a signigicantly stronger correlation than the low ratings.

X = Contrary to the hypothesis, the high ratings for the painting have a significantly lower correlation than the low ratings.

D = As hypothesized, the high ratings are greater than low ratings in the correlation coefficients at the third second.

X = Contraty to the hypothesis, the high ratings are greater than the low ratings in the correlation coefficients at the third second. Gray shading highlights the “X” cases for easier visual recognition.

Low rated.

✓ = As hypothesized, the low ratings for the painting show a signigicantly stronger correlation than the high ratings.

X = Contrary to the hypothesis, the low ratings for the painting have a significantly lower correlation than the high ratings.

D = As hypothesized, the high ratings are greater than low ratings in the correlation coefficients at the third second.

X = Contraty to the hypothesis, the high ratings are greater than the low ratings in the correlation coefficients at the third second. Gray shading highlights the “X” cases for easier visual recognition.

Case for “favourability” (Table 6)

- 5 highly-rated (Great Wave, Mannenbashi, Ejiri, Futagawa, Shinagawa): CVB in Figure 7 > CVB in Figure 8 → Consistent with the hypothesis.
- 7 low-rated (Okazaki, Ryogokubashi, Kazusa, Okitsu, Fujikawa, Fujieda, Akasaka): CVB in Figure 7 < CVB in Figure 8 → Consistent with the hypothesis.

**Table 6:** Normalised “Favourability” and correlation of VH-LL and “Favourability.”

	Title	Normalised ‘Favorability’					*		Correlation of VH-LL and ‘Favorability’																	
		Rank	Overall Average				3		6		9		12		15		18		21		24		27		30	
				High	Middle	Low	G1	G2	G1	G2	G1	G2	G1	G2	G1	G2	G1	G2	G1	G2	G1	G2	G1	G2	G1	G2
Highly rated	Under the Wave off Kanagawa Commonly known as ‘Great Wave’	**1**	**0.94**	1.76	1.00	0.05	D	D			✓		✓	✓	✓	✓	✓	✓	✓	✓	✓	✓	✓	✓	✓	✓
	Under the Mannenbashi Bridge at Fukagawa	**2**	**0.35**	1.28	0.49	−0.71	D																			
	Ejiri in Suruga Province	**3**	**0.33**	1.29	0.46	−0.74	X	X			X	X	X	X	X	X	X	X	X	X	X	X	X	X	X	X
	Futagawa: Sarugababa	**4**	**0.10**	1.27	0.23	−1.20	X	X			X	X	X	X	X	X	X	X	X	X	X	X	X	X	X	X
Low rated	Shinagawa: Sunrise	**5**	**−0.07**	0.90	0.01	−1.12	X	X			X	X	X	X	X	X	X	X								
	Akasaka: Inn with Serving Maids	**6**	**−0.13**	0.81	−0.04	−1.14	X	X			✓	✓	✓	✓	✓	✓	✓	✓	✓	✓	✓	✓	✓	✓	✓	✓
	Okazaki: Yahagi Bridge	**7**	**−0.29**	0.79	−0.41	−1.26	X	X																		
	Viewing Sunset over the Ryogokubashi Bridge from the Ommayagashi River Bank	**8**	**−0.34**	0.75	−0.35	−1.41	X	X								✓		✓	✓	✓	✓	✓	✓	✓	✓	✓
	Sea Lane off Kazusa Province	**9**	**−0.34**	0.70	−0.32	−1.40	X	X			X	X	X	X	X	X	X	X	X	X	X	X	X	X	X	X
	Okitsu: The Okitsu River	**10**	**−0.40**	0.47	−0.44	−1.22	X	X					X	X	X	X	X	X	X	X	X	X	X	X	X	X
	Fujikawa: Scene at the Boundary Marker	**11**	**−0.69**	0.44	−0.82	−1.70	X	X	X	X			X		X	X	X	X	X	X	X	X	X	X	X	X
	Fujieda: Changing Porters and Horses	**12**	**−0.79**	0.40	−0.91	−1.86	D	D			✓	✓	✓	✓	✓	✓	✓	✓	✓	✓	✓	✓	✓	✓	✓	✓

Highly rated.

✓ = As hypothesized, the high ratings for the painting show a signigicantly stronger correlation than the low ratings.

X = Contrary to the hypothesis, the high ratings for the painting have a significantly lower correlation than the low ratings.

D = As hypothesized, the high ratings are greater than low ratings in the correlation coefficients at the third second.

X = Contraty to the hypothesis, the high ratings are greater than the low ratings in the correlation coefficients at the third second. Gray shading highlights the “X” cases for easier visual recognition.

Low rated.

✓ = As hypothesized, the low ratings for the painting show a signigicantly stronger correlation than the high ratings.

X = Contrary to the hypothesis, the low ratings for the painting have a significantly lower correlation than the high ratings.

D = As hypothesized, the high ratings are greater than low ratings in the correlation coefficients at the third second.

X = Contraty to the hypothesis, the high ratings are greater than the low ratings in the correlation coefficients at the third second. Gray shading highlights the “X” cases for easier visual recognition.

### 2.7 Data analysis for pupil size

Following previous studies such as the one conducted by Nuthmann and van der Meer (2005), we analysed pupil size as a measure of processing fluency. Because processing fluency is reflected in pupil size, and if pupil changes are primarily the result of cognitive processing, the most pronounced pupil constrictions would be observed under conditions of high processing fluency and the shortest reaction times. Therefore, we analysed the time-series changes in pupil size based on the hypothesis that the higher the processing fluency, the more pupil constriction and the higher the aesthetic evaluation. Since pupil responses have been shown to appear approximately 300 ms after stimulus onset (Gao et al., 2020), we determined that no adjustment for the time difference between responses was necessary in this study.

**Figure 9:**
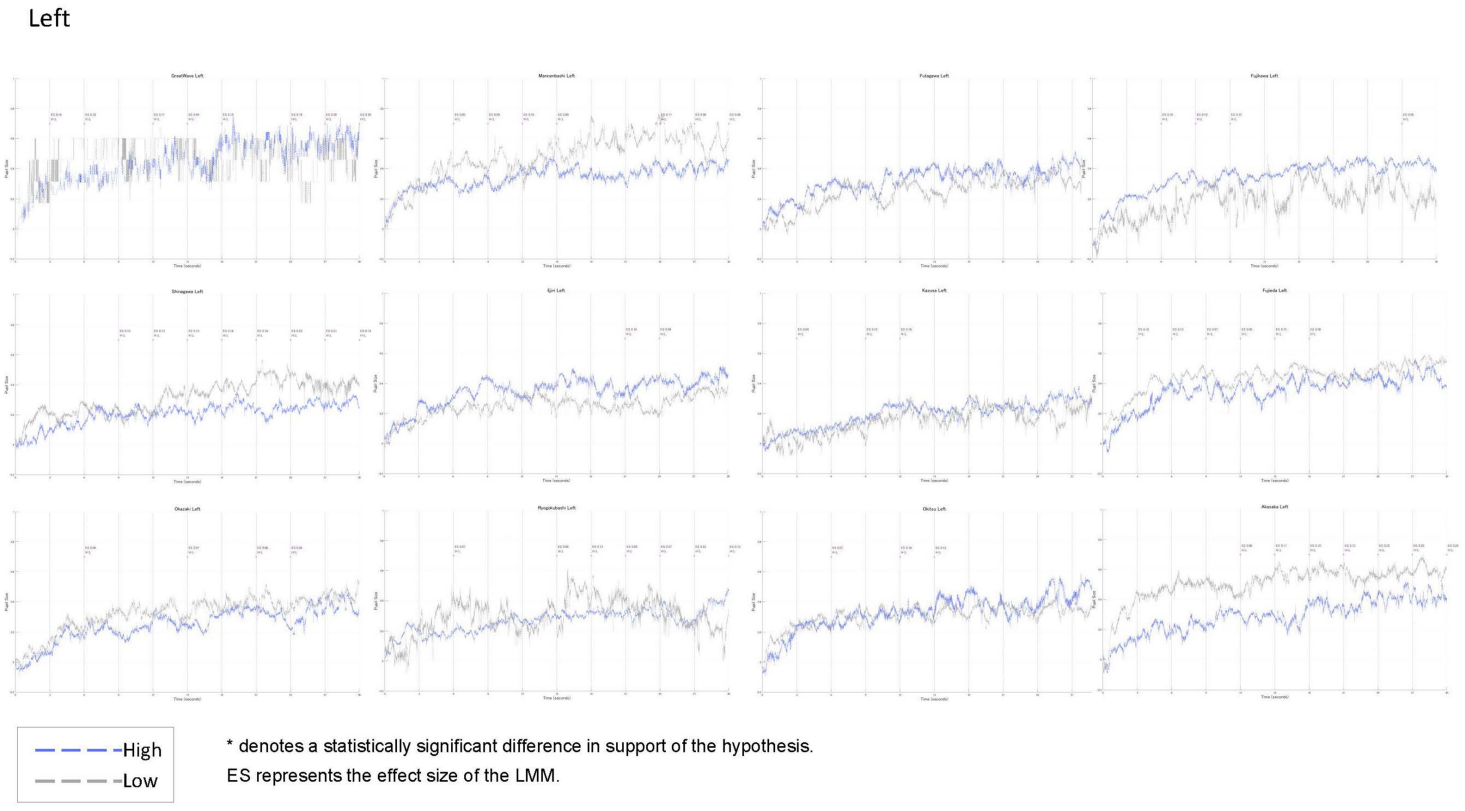
Time-series changes in pupil size (Left eye). Blue lines represent the high-evaluation group, and grey lines represent the low-evaluation group. Pupil size (0–30 s, sampling rate 300 Hz) is plotted on the horizontal axis, with normalised values (−0.2 to 1.0) on the vertical axis. Asterisks indicate time points at which the high-evaluation group showed significantly smaller pupil size than the low-evaluation group, supporting the hypothesis that higher processing fluency (greater aesthetic appreciation) is associated with stronger pupil constriction.

**Figure 10:**
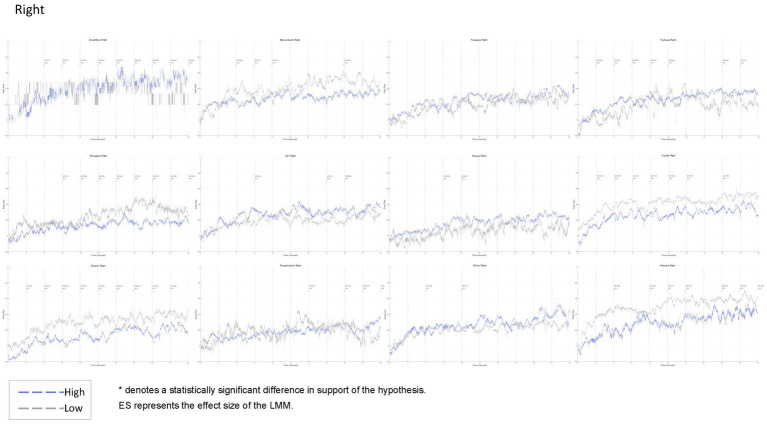
Time-series changes in pupil size (Right eye). Blue lines represent the high-evaluation group, and grey lines represent the low-evaluation group. Pupil size (0–30 s, sampling rate 300 Hz) is plotted on the horizontal axis, with normalised values (−0.2 to 1.0) on the vertical axis. Asterisks indicate time points at which the high-evaluation group showed significantly smaller pupil size than the low-evaluation group, consistent with the hypothesis that enhanced processing fluency corresponds to greater pupil constriction and higher aesthetic evaluation.

Using Mathôt and Vilotijević (2023), the uncorrected mean of the pupil size of the cross rest screen on a white background presented 5 s before the stimulus was used as a baseline, and the pupil size of each participant for 30 s was subtracted. Outliers were excluded using the 3-sigma method, and missing data were linearly corrected with 50 before and after data using the fillmissing function provided by Matlab R2023a. K-means clustering was applied to the normalised “beauty” to classify participants into three groups: high, middle, and low. This approach enabled us to identify relative differences in image evaluations and to capture overall evaluation trends. Pupil size of the high and low rated participants in the three groups, left and right separately, were tested in 3 s increments under the hypothetical assumption that the high rated group would have smaller pupil size. Because of the finding that aesthetic evaluation takes place by the sixth second of initial viewing obtained from a previous study, testing for the entire 30 s may miss an important early-stage response. For this reason, we analysed changes over a short period of time, including pupil size in the initial phase. In this case, even when the LMM was conducted in 3 s increments, the cumulative changes at each time point were tracked in the form of “0 to 3 s” and “0 to 6 s” to maintain treatment as a continuous variable. Finally, based on the hypotheses, we tested the significance of the pupil size of the participants in the high and low evaluation groups in the three groups. Since the number of samples in this experiment varied depending on the photograph and evaluation, the LMM was used to test the hypotheses. The number of seconds for which there was a significant difference, supporting the hypothesis that “the highly rated group had smaller pupil size than the low rated group,” is indicated by an asterisk and effect size on the graph. Pupil size from 0 to 30 s (sampling rate 300) on the horizontal axis and −0.2 to 1.0 on the vertical axis are shown in Figure 9 (Left) and Figure 10 (Right), with the blue line representing the high-evaluation group and the grey line representing the low-evaluation group.

## 3 Results

We tested the RAIC hypothesis—that compositionally guided viewing predicts higher aesthetic appraisal—using three complementary analyses: (i) gaze–rating link, quantifying for each image the VH-LL (log-likelihood of each participant’s scanpath under the EGP, the experts’ gaze patterns) and its correlation with beauty ratings (CVB); (ii) verbal content, probing RAIC effects with CART-informed coding of verbal responses; and (iii) processing fluency, indexing perceptual effort with pupil metrics. Across images, beauty showed stronger and earlier RAIC effects than favourability, with the clearest divergences within the first 6 s (notably Great Wave, Ejiri, Ryogokubashi, Futagawa, Okitsu, Fujikawa, and Akasaka). Pupil analyses converged, indicating greater fluency at higher beauty.

### 3.1 CVB summary

Table 5 summarises the results of statistical testing for differences in CVB between the high- and low-evaluation groups of “beauty.” At 3 s, 9 of the 12 paintings (excluding Shinagawa, Ejiri, and Fujikawa) showed a pattern consistent with the hypothesis, and significant differences (*p* < 0.05) were observed in four paintings at 6 s (Ryogokubashi, Futagawa, Okitsu, Akasaka), in Great Wave at 9 s, and again in Akasaka at 15 s (Table 2). However, several works—notably Shinagawa, Ryogokubashi, Okazaki, Fujieda, and Kazusa—showed patterns that frequently diverged from the hypothesised relationship during the 30 s viewing period.

Table 6 presents the results of statistical tests for significant differences in CVB related to “favourability” between the high and low evaluation groups, using the same procedure as that applied for “beauty.” The high evaluation group included five paintings (Great Wave, Mannenbashi, Ejiri, Futagawa, and Shinagawa), while the low evaluation group included seven paintings (Akasaka, Okazaki, Ryogokubashi, Kazusa, Okitsu, Fujikawa, and Fujieda). Notably, compared to the grouping for “beauty,” Futagawa was classified as high-rated, while Okazaki and Ryogokubashi appeared in the low-rated group.

When comparing normalised impression scores, the difference between the highest-rated painting (Great Wave, 0.74) and the second highest (Shinagawa, 0.21) in the high-evaluation group was 0.53, suggesting a steep decline in ratings after the top-ranked work. By contrast, in the low-evaluation group the gap between the lowest-rated (Akasaka, −0.54) and the second lowest (Fujikawa, −0.50) was only 0.04, with a smaller standard deviation (SD = 0.20). Although the paintings were selected from a pilot study of 101 works (top six vs. bottom six), the difference between the high- and low-rated groups in this experiment was less pronounced. This attenuation may reflect contrast effects caused by sequential stimulus presentation or variability in participants’ reference frames, whether pre-existing or constructed dynamically during viewing. Even under these conditions, Great Wave consistently maintained a distinct evaluation gap between groups, marking it as the only work in the set to show a stable divergence in impression ratings across time.

Painting-wise results were as follows:

- Great Wave: Strong CVB Consistency; participants’ gaze paths aligned with expert guidance throughout, sustaining a stable evaluation gap.
- Shinagawa: Minimal CVB Consistency; alignment with expert-defined gaze patterns was weak, and impression scores showed little correspondence.
- Okazaki: Moderate CVB Consistency; explorative strategies dominated, but closer gaze alignment did not yield higher ratings.
- Mannenbashi: Strong CVB Consistency; focused strategies centred on key foreground ROIs, producing clear differences between groups.
- Ejiri: Moderate CVB Consistency; correlations were negative in early viewing, with divergence from expert paths linked to higher ratings.
- Ryogokubashi: Moderate CVB Consistency; initially consistent but weakened over time as gaze remained fixed on a single ROI.
- Futagawa: Strong CVB Consistency; inverse correlations consistent with its sombre theme supported the hypothesis.
- Kazusa: Minimal CVB Consistency; strong visual salience of the ship constrained exploration, keeping ratings low.
- Okitsu: Strong CVB Consistency; stronger alignment with expert gaze patterns in the low-rated group matched the hypothesis.
- Fujikawa: Strong CVB Consistency; closer adherence to expert gaze in the low-rated group reinforced negative impressions.
- Fujieda: Minimal CVB Consistency; gaze was strongly focused, but alignment with expert paths did not enhance ratings.
- Akasaka: Strong CVB Consistency; consistent alignment with expert guidance was observed, in line with the hypothesis.

Paradoxical cases. Notably, in several prints—most clearly Okitsu, Fujikawa, and Akasaka (and in early intervals for Ejiri)—the low-rated group exhibited equal or greater alignment between participant and expert gaze patterns. This suggests that compositionally guided viewing can also intensify the salience of unsettling or socially charged content, yielding lower beauty despite stronger guidance. In other words, RAIC appears to enhance the clarity of the intended message, not necessarily its valence.

### 3.2 Processing fluency measured by pupil size

Figures 9, 10 and Table 7 show the results of the pupil size analysis for the high- and low-evaluation groups, classified via k-means clustering based on normalised beauty ratings.

**Table 7:** The average pupil size for the high and low rated groups of “Beauty.”

	Title	Pupil size																			
		3		6		9		12		15		18		21		24		27		30	
		L	R	L	R	L	R	L	R	L	R	L	R	L	R	L	R	L	R	L	R
Highly rated	Under the Wave off Kanagawa Commonly known as ‘Great Wave’	0.16		0.18	0.17		0.18	0.11	0.18	**0.44**	**0.32**	0.10	0.18		**0.26**	0.19	**0.38**	**0.20**	**0.32**	**0.30**	**0.41**
	Shinagawa: Sunrise					0.10	0.11	0.13	0.08	0.13	0.15	0.18	**0.22**	0.18	0.17	**0.23**	**0.19**	**0.21**	**0.26**	0.19	**0.24**
	Ejiri in Suruga Province						0.06							0.07	0.05	0.07	0.12		0.06		
	Under the Mannenbashi Bridge at Fukagawa							0.08	0.14	0.10	0.07	0.09								0.12	0.09
	Viewing Sunset over the Ryogokubashi Bridge from the Ommayagashi River Bank		0.05	0.07	0.05				0.06			0.10	0.12	0.07	0.05	0.09	0.16	**0.23**	**0.20**	0.14	0.11
	Okazaki: Yahagi Bridge		0.11	0.06	0.18		0.16		0.10	0.07	0.16		0.18	0.06	0.15	0.08	0.15		0.09		
Low rated	Futagawa: Sarugababa														0.06						
	Fujieda: Changing Porters and Horses	0.10	0.19	0.13	0.18	0.07	0.15	0.05	0.10	0.12	0.15	0.06	0.10			0.06					
	Sea Lane off Kazusa Province	0.04				0.12	0.08	0.16	0.12												
	Okitsu: The Okitsu River	0.04		0.05	0.07			0.14	0.11	0.13				0.07							
	Akasaka: Inn with Serving Maids				0.06			0.08	0.07	0.17	0.17	0.10	0.12	0.12		**0.22**	0.15	**0.25**	**0.24**	**0.22**	**0.21**
	Fujikawa: Scene at the Boundary Marker	0.08	0.14	0.14	**0.23**	0.10	0.13		0.06	0.05	0.11					0.06					
Significant difference (H0 support)		5	4	6	7	4	7	7	10	8	7	6	6	6	6	8	6	4	6	5	5

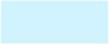
 = Middle level of the effect size (>0.30).

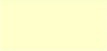
 = Small level of the effect size (>0.20).

Pupil data were sampled at 300 Hz, and the mean pupil sizes for each group are plotted in blue and grey, respectively. To test the hypothesis that “higher aesthetic evaluations are associated with smaller pupil sizes,” a linear mixed-effects model (LMM) was applied. Time points showing statistically significant differences (*p* < 0.05) in line with the hypothesis are marked with asterisks, with corresponding effect sizes indicated above the graph. Medium effect sizes (Cohen’s *d* > 0.30) are shown in blue; small effects (*d* > 0.20) are indicated by yellow hatching.

In total, 11 of the 12 artworks (excluding Futagawa) showed statistically significant differences supporting the hypothesis, primarily between 3 and 30 s after stimulus onset, although most of these effects were small (*d* < 0.20). Notably, Great Wave exhibited a medium-sized effect after the 15 s mark. This artwork also showed the greatest within-group variability in pupil size for the high-evaluation group (mean SD = 0.15), substantially above the overall average (SD = 0.10).

Such delayed and pronounced pupil responses may reflect not early perceptual processing, but later-stage cognitive mechanisms such as semantic integration or interpretive evaluation. The high degree of variation observed Great Wave suggests that viewers experienced a broader range of perceptual or emotional responses than for other artworks.

### 3.3 RAIC effect

The results of RAIC’s analysis are shown below, referring to the CART theory as explained in section 2 Materials and Methods.

**Great Wave; Strong RAIC Effect** (1) Great Wave was a particularly clear example of RAIC, successfully guiding viewers through a structured visual narrative. This work is widely recognised for its dynamic composition and strong narrative quality, depicting long, narrow fishing boats struggling against immense waves. It employs a heterochronous composition, in which sequential motion is captured within a single frame, thereby producing a visual flow that spans both space and time (Kato and Ota, 2007). The composition guides the viewer’s gaze naturally from the boats in the foreground (ROI1, ROI2) towards the distant offshore area (ROI13), creating a cinematic effect (Thompson et al., 2014, p. 194). This structured visual pathway is thought to facilitate intuitive narrative comprehension and enable viewers to reconstruct the artist’s intended story. As a result, the RAIC effect appeared particularly strong: in a pilot study, 34 out of 48 participants rated the image as “very beautiful and desirable.” These findings suggest that clearly defined compositional cues not only guide eye movements, but may also enhance the viewer’s overall aesthetic experience.

**Shinagawa; Limited RAIC Effect** (1) Only 4 out of 18 participants who rated the painting highly made metaphorical remarks such as “I did not know Shinagawa used to be so lively” or “lively and peaceful,” indicating weak narrative reconstruction. Historically, Shinagawa was the first post town on the Tokaido Highway and is depicted as the beginning of the journey, featuring the lead of a daimyo procession and a prominent inn (Utagawa, 1991, p. 114). However, the majority of responses (13 out of 18) were literal impressions such as “good colouring of the sky” (9 cases) and “well-balanced composition” (3 cases), with little reference to story or intentionality. The composition includes finely detailed depictions of people and architecture, but the gaze patterns tended to circulate locally around prominent foreground elements. This suggests that visual attention was more focused on individual pictorial features than on sequential narrative flow, possibly limiting the effectiveness of RAIC.

**Okazaki; Limited RAIC Effect** (2) Despite some narrative interpretations, the RAIC effect was limited, likely due to the small visual prominence of key elements in the composition. Nevertheless, 6 out of 18 high-rating comments included metaphorical interpretations, suggesting that some viewers were able to construct a narrative. For example, several participants inferred a story such as: “The small castle at the end of the bridge is probably the destination of this group, and I think they are all relieved.” This scene is set on the longest bridge along the Tokaido Highway, depicting a procession nearing arrival at Okazaki, historically the second most prosperous post town (Utagawa, 1991, p. 121). However, because the people and castle are relatively small within the frame, the scene’s intent may not have been immediately apparent. This lack of clarity likely contributed to stronger localised gaze behaviour and a lower match between EGP and PGP.

**Mannenbashi; Strong RAIC Effect** (2) Of the 18 participants who rated this image highly, 7 offered metaphorical interpretations—such as “quietness,” “as if I am peeking out from under the bridge,” and “bustling above the bridge and calm below”—while another 8 praised literal elements like colour, symmetry, and depth. This work depicts a tranquil, horizontally expansive landscape featuring a prominent drum bridge spanning the composition, with townspeople crossing it, samurai residences on either bank, and Mt. Fuji visible in the distance. According to Yanagi (1957), the bridge functions compositionally like a telescope, naturally directing the viewer’s gaze towards Mt. Fuji on the horizon. The high degree of alignment between EGP and PGP (Table 2) suggests that the artist effectively conveyed narrative intention through compositional cues. This strong RAIC effect likely contributed to the painting’s overall aesthetic impact.

**Ejiri; Limited RAIC Effect** (3) Despite receiving high aesthetic ratings, Ejiri exhibited low CVB values, indicating that participants’ gaze patterns did not align with EGP. Nevertheless, 12 participants in the high-rating group provided metaphorical responses, many of which described the force of the invisible wind or conveyed a sense of immersion, as if they were physically present in the scene. This suggests that, even without gaze trajectories matching the ROI-defined path, the composition itself may have sufficiently facilitated perceptual and emotional engagement with the narrative. CVB analysis further revealed a tendency in both groups to initiate their gaze near the centre of the image (ROI8), which corresponds to the vanishing point. As noted by Yanagi (1957), this work features a clear vanishing point and strong vertical symmetry, implying that gaze guidance may have been shaped more by spatial depth than by EGP. This perceptual bias towards central elements likely enhanced the sense of immersion and narrative comprehension. Taken together, these findings suggest that in Ejiri, the RAIC effect may operate through a dual mechanism: one based on narrative guidance via ROI-defined transitions, and another rooted in spatial immersion created by depth and central composition.

**Ryogokubashi; Minimal RAIC Effect** (1) Ryogokubashi received primarily literal impression ratings, with six out of 14 high-rating participants commenting on the aesthetic quality of the wave patterns. Additionally, two participants made metaphorical statements describing the scene as evoking a sense of motion and a narrative of workers finishing their day and boarding a small boat to enjoy the sunset and a distant view of Mt. Fuji. Notably, the painting’s title was not disclosed to participants in order to avoid influencing their impression ratings. As a result, seven of the 48 viewers described the darkened sky as “creepy,” suggesting that the absence of contextual information may have negatively affected their interpretations. From a visual composition perspective, the elaborately rendered wave patterns in the foreground may have attracted early fixations, resulting in a starting point that deviated from EGP. This suggests a possible *anchoring effect of early fixations*, whereby the initial focus of attention biased subsequent gaze behaviour. Such anchoring may have disrupted the intended sequential viewing path and limited narrative reconstruction. Overall, while some metaphorical engagement was observed, the RAIC effect remained minimal for this work.

**Futagawa; Strong RAIC Effect** (3) Futagawa features a restrained composition dominated by subdued dark greens, depicting two rounded hills, three women beggars, a roadside tea stall, and a sparsely rendered field (Utagawa, 1991, p. 120). The arc-like contour of the hills appears to guide the viewer’s gaze across the image, indicating deliberate visual structure. However, among the 14 participants in the low-rated group, many responses were metaphorical or negatively toned, such as “the trees feel sparse and barren,” “lonely,” and “painted without care.” Literal responses also conveyed confusion, including “I cannot tell what this depicts” and “I do not know where the depth is.” These impressions suggest that, despite coherent composition, the minimal detailing and stylized spatial rendering characteristic of *Ukiyo-e* may have hindered deeper narrative engagement. Taken together with the CVB findings, these results imply that RAIC in Futagawa may have contributed not only to aesthetic evaluation, but also to the successful communication of emotionally sombre or discomforting content. RAIC, in this case, may not have enhanced beauty per se, but rather served to strengthen the expressive impact of a restrained narrative through structured compositional guidance.

**Kazusa; Minimal RAIC Effect** (2) In terms of RAIC, Kazusa appeared to evoke limited narrative understanding. Among the low-rated group, 10 out of 16 participants provided literal comments such as “the ship is not moving,” “it’s hard to focus,” and “the sense of depth feels unnatural.” Others offered metaphorical remarks like “it looks windy” or “it seems childlike,” indicating difficulty in engaging with the scene’s intended meaning or depth. The composition, influenced by Hokusai’s Dutch-inspired maritime sketches, emphasised the dramatic form of the vessel seen from a low angle rather than presenting a cohesive narrative structure. While compositional cues such as the horizon line and deck edge may have been intended to guide the viewer’s gaze (Thompson et al., 2014, p. 195), the dominance of the ship’s hull likely disrupted this guidance. This visual imbalance may have impeded the reconstruction of spatial depth and narrative flow. According to research in visual cognition, such reconstruction relies on the comparison of visual input with internal reference models based on prior experience (Koenderink, 1990; Palmer, 1999). In Kazusa, the reconstruction process may not have worked effectively for some viewers, which might have made it more difficult to interpret the artist’s intention and thus contributed to a weaker RAIC effect.

**Okitsu; Strong RAIC Effect** (4) Okitsu serves as a compelling example in which visual composition effectively conveys a socially oriented narrative, making it particularly insightful from the perspective of RAIC. The image depicts five underdressed labourers carrying a high-ranking individual across a river in a palanquin—a striking scene of social hierarchy and physical burden. Among the 14 participants in the low-rated group, seven provided metaphorical impressions such as “I feel the status difference,” “I feel sorry for the labourers,” and “The samurai seem to be great,” reflecting affective engagement with the characters’ social roles. Literal evaluations focused on compositional elements, especially the foreground rock, with comments like “The rock stands out too much” or “I could not look at anything else.” This rock (ROI6) was among the most frequently fixated areas and showed high transition probabilities, indicating strong visual guidance. These responses, together with the high CVB alignment observed particularly in Group 1, suggest that the visual composition successfully directed the viewer’s attention in a way that highlighted the social implications embedded in the scene. Thus, while the aesthetic evaluation was low, the image appears to have fulfilled a narrative function through its compositional cues, underscoring the potential of RAIC to enhance the transmission of socially charged meaning, even in the absence of high aesthetic appeal.

**Fujikawa; Strong RAIC Effect** (5) Fujikawa presents a compelling example in which highly effective gaze guidance may have accentuated the painting’s negative narrative content, thereby contributing to lower aesthetic evaluations. The image depicts townspeople bowing deeply before a feudal lord’s procession, with even a small dog in the foreground appearing to bow as well. Despite this humorous detail, many participants described the overall impression as “dark” or found the content difficult to interpret. Among the 16 participants in the low-rating group, five provided literal evaluations related to the composition, citing “uncomfortable colours” or “unnatural perspective.” Two others offered imaginative comments, such as “the colours must have originally looked different,” suggesting an interpretive engagement that considered the potential effects of colour degradation over time. As supported by the CVB analysis, PGP showed high alignment with EGP, indicating a high RAIC effect. However, this strong visual guidance may have heightened viewers’ awareness of the painting’s socially charged or emotionally ambiguous themes, ultimately reinforcing a negative impression. Thus, in this case, successful narrative reconstruction through gaze guidance did not lead to a more positive aesthetic experience.

**Fujieda; Minimal RAIC Effect** (3) Although the image was highly structured in terms of composition—divided into three horizontal bands and featuring diagonal lines praised by Yamaguchi (1981)—the narrative clarity perceived by viewers remained limited. Among the 18 participants in the low-rated group, four provided metaphorical responses. These included impressions such as “the labourers seem to be having a hard time” or “the scene feels busy but disjointed,” suggesting emotional engagement but not necessarily narrative coherence. In addition, four literal comments focused on discomfort with the colour palette and spatial depth, noting that “the hues feel mismatched” and “the perspective seems awkward.” The painting depicts a group of labourers (*ninsoku*)—shirtless men performing manual work—stretching across the scene in a diagonally receding line. The foreground is rendered in three colour tones (green, yellow, and brown), contributing to spatial segmentation. Despite these compositional cues, participants’ gaze behaviour, as previously described in the CVB analysis, remained highly focused on specific figures (particularly ROI2), and did not align with EGP. This suggests that while the visual structure may have evoked some affective responses, it did not effectively support viewer reconstruction of the artist’s intended story—an essential criterion for strong RAIC.

**Akasaka; Strong RAIC Effect** (6) Akasaka illustrates how effective visual guidance can elicit emotionally charged responses when the underlying narrative is morally or psychologically complex. Among the 15 participants in the low-rated group, seven provided metaphorical and emotionally charged comments. These included statements such as “I feel guilty, as if I’m peeping in” (*n* = 4), “It’s scary” (*n* = 1), and “I do not understand what this painting is about” (*n* = 1). Additionally, one participant offered a literal comment noting that “the tree in the foreground disrupts the composition.” The painting depicts a brothel’s veranda as seen from the outside during twilight. A large, dark palm-like tree (ROI1) occupies the centre of the frame, and its spiral shape, central placement, and proximity to subsequent ROIs serve as strong visual guides that naturally direct the viewer’s gaze through the intended sequence. As shown in the CVB analysis, PGP more closely adhered to EGP tended to give lower impression ratings. This suggests that the composition effectively directed attention to morally or emotionally sensitive content, facilitating narrative reconstruction in a way that ultimately overwhelmed the aesthetic experience, despite the successful activation of RAIC.

## 4 General discussion

### Greater RAIC success leads to a more profound aesthetic experience

Based on the findings of Locher et al. (2008), this study hypothesised that the more a viewer’s gaze aligns with that of an expert during art appreciation, the more likely the RAIC (Reading an Artist’s Intention from the Composition) will be established, thereby enhancing the understanding of the artwork’s compositional world and intensifying the aesthetic experience. The experimental results provided partial support for this hypothesis. Specifically, a higher degree of similarity (CVB) between expert gaze patterns (EGP) and participant gaze patterns (PGP) was associated with higher aesthetic ratings. Moreover, in artworks where gaze guidance was considered to have been effective and RAIC successfully established, participants exhibited more consistent evaluations, suggesting that the artist’s intention and compositional structure had been adequately conveyed.

In particular, the claims by Locher et al. (2008) that “the smoothness of eye movements” and “the visual fluency of compositional elements” contribute to aesthetic judgement were in line with the present findings. When RAIC functioned successfully, viewers found it easier to comprehend the artist’s intention. Depending on whether the narrative content of the work was perceived as positive or negative, aesthetic evaluations tended to increase or decrease, respectively. Compositional gaze guidance often led to the reconstruction of the artwork’s narrative or social message, which in turn elicited stronger emotional and ethical reactions. For instance, artworks with an emphasised positive storyline (e.g., Great Wave, Mannenbashi) received higher ratings, whereas those portraying socially weighty themes (e.g., Fujikawa, Akasaka) evoked emotional responses such as moral indignation, pressure, and discomfort, ultimately resulting in lower evaluations. These findings are consistent with cognitive psychological perspectives, such as those of Reber et al. (2004), which argue that the fluency of visual processing is linked to emotional pleasure or displeasure and can amplify aesthetic experience.

In addition, the establishment of RAIC appears to involve not only the physical synchronisation of gaze patterns but also the viewer’s ability to engage in contextual and metaphorical interpretation. The results suggest that higher similarity between EGP and PGP may indicate successful RAIC. Notably, when verbal impressions included metaphorical or context-dependent elements, the RAIC effect tended to be stronger. This relates to the broader question of how metaphorical impressions are formed. While *Ukiyo-e* may appear realistic and direct in depiction, symbolic or metaphorical meanings may be drawn from them through the viewer’s interpretation. As Ernest Fenollosa noted (Fenollosa, 1892), *Ukiyo-e* artists “consciously discarded all literary, religious, moral, and aesthetic ideals, and without striving for realism, undertook to reflect popular trends and vulgar entertainments in terms accessible to the common people.” In this sense, *Ukiyo-e* can be considered an art form that transcends realism, inviting viewers to imagine and engage with everyday scenes.

Indeed, among the 12 prints analysed in this study, some clearly conveyed metaphorical meanings, while others appeared superficially realistic but invited contextual interpretation through the viewer’s imagination. Given this structural nature, it is difficult to define the success of RAIC purely in terms of perceived beauty. Rather, it may be the process itself—whereby the viewer seeks to interpret the work and connect with the artist’s intent—that lies at the heart of RAIC.

Regarding the temporal dimension of gaze behaviour, the early phase (3–9 s) was found to be strongly associated with aesthetic judgement. It is presumed that the RAIC effect plays a role in this early stage by shaping impression formation. Future studies incorporating neuroscientific techniques such as fMRI are expected to further clarify the causal relationship between visual attention and emotional evaluation.

The effect of RAIC could not be explained by a simple linear relationship with narrative understanding based on ROI (Regions of Interest), suggesting that a multilayered interpretive process is at work. Depth perception and the ability to reconstruct spatial immersion were also found to influence aesthetic evaluation. For instance, although compositional guidance was present in Kazusa, the difficulty in reconstructing spatial depth may have contributed to its low rating. In contrast, Ejiri was likely rated more favourably due to the powerful pull of its vanishing point (ROI4), which enhanced immersion, and the presence of motifs that implicitly evoked wind, fostering imaginative engagement. Although Futagawa exhibited high CVB values indicating effective gaze guidance, its insufficient spatial information likely hindered narrative understanding, leading to lower ratings. These cases suggest that even when RAIC is established, depth perception and spatial coherence may act as mediating variables in aesthetic judgement.

The relationship between RAIC and aesthetic evaluation is not strictly linear; instead, it appears to vary depending on how emotionally the reconstructed narrative or space is received. In landscape prints, as opposed to portraits, the composition more directly contributes to a sense of immersion and presence, which may accentuate this effect. Conversely, in artworks where the RAIC effect was limited, features unique to *Ukiyo-e*—such as distortions in perspective or fading pigments (Kadar and Effken, 2008)—may have disrupted visual consistency and hindered spatial reconstruction. Using digitally restored versions reflecting the original colours may allow for clearer identification of RAIC effects.

Furthermore, physiological evidence—particularly pupil diameter—was used to examine the relationship between aesthetic judgement and perceptual fluency. Pupil constriction was observed in highly rated artworks, suggesting that it may serve as a physiological index of perceptual fluency and emotional engagement. This finding aligns with the James-Lange theory of emotion, which posits that emotional responses arise from bodily reactions. However, further neuroscientific research is needed to determine whether pupil constriction precedes the recognition of beauty or follows as a result of evaluation.

Two distinct gaze patterns were identified among expert viewers associated with high RAIC effects: one involving circular or spiral movement across the entire image (e.g., Great Wave, Fujieda, Akasaka), and another following a Z-shaped path from the bottom to the top of the image (e.g., Okazaki, Okitsu). In the latter pattern, the initial central fixation point during the experiment may have disrupted natural gaze movement, suggesting that future studies should consider refining the presentation method.

This study, building on the prior work of Belfi et al. (2019) and Locher et al. (2008), empirically investigated how specific gaze patterns contribute to impression formation. The primary contributions of this study are as follows:

(1) When RAIC is successfully established through compositional gaze guidance, viewers tend to better understand the artist’s intention, and aesthetic evaluation rises or falls depending on the positivity or negativity of the narrative content.
(2) Gaze behaviour in the first 3 s plays a critical role in impression formation.
(3) A statistically significant relationship was observed between pupil constriction and higher evaluations, supporting the link between perceptual fluency and aesthetic judgement.

These findings offer a novel framework for understanding the interaction between gaze behaviour and physiological responses in art appreciation, integrating cognitive and emotional processes.

### Limitations and future directions

The paradoxical cases indicate that RAIC does not guarantee higher beauty per se; rather, stronger alignment between expert-defined and participant scanpaths may amplify the perceived intent of a work, including morally or affectively adverse narratives. Methodologically, our alignment metric emphasised transition probabilities among expert-defined ROIs; it did not directly model dwell-time weighting or re-visit patterns, and participants began each trial from a central fixation, which can bias early trajectories. Sequential presentation may also have introduced contrast effects, and per-image subgroup sizes limited power in some comparisons. Future work should (i) treat narrative valence as a moderator of the RAIC–evaluation link; (ii) compare multiple sequence-sensitive similarity measures (e.g., order-based and time-warped metrics) alongside transition-based indices; (iii) counterbalance or randomise presentation sequences with preregistered analyses; and (iv) include explicit ratings of perceived authorial intent and affect to test whether RAIC primarily increases interpretive clarity rather than favourability.

Lastly, this study utilised landscape prints from the *Ukiyo-e* genre as visual stimuli. *Ukiyo-e*, being woodblock prints intended for mass reproduction, served as popular media in Edo-period Japan to convey secular (‘*Ukiyo*’ in Japanese) themes. As artworks designed to communicate information, they are well suited to RAIC-based analysis. In the future, applying this framework to digitally restored first-edition prints or other genres will help assess both the generalisability and the limitations of the RAIC approach.

## Data Availability

All data and materials (i.e., fixation data, pupil size, impression evaluation value, analysis code) are publicly available via the Open Science Framework and can be accessed at https://osf.io/uzhgv/?view_only=cdf46badd8ea4b9398dc20b3ed2def05.
