# Correction: Reading an Artist's Intention from the Composition (RAIC): eye movements and aesthetic experience in Japanese woodblock prints

**DOI:** 10.3389/fpsyg.2025.1758405

**Published:** 2026-01-27

**Authors:** Frontiers Production Office

**Affiliations:** Frontiers Media SA, Lausanne, Switzerland

**Keywords:** eye movements, aesthetic perception, gaze patterns, a variational Bayesian hierarchical extension of the EMHMM (VBHEM), Japanese woodblock prints (*Ukiyo-e*)

There was a mistake in Figure 1 as published. The red arrows were missing from the Gaze Guided image. The corrected Figure 1 appears below:

**Figure 1:**
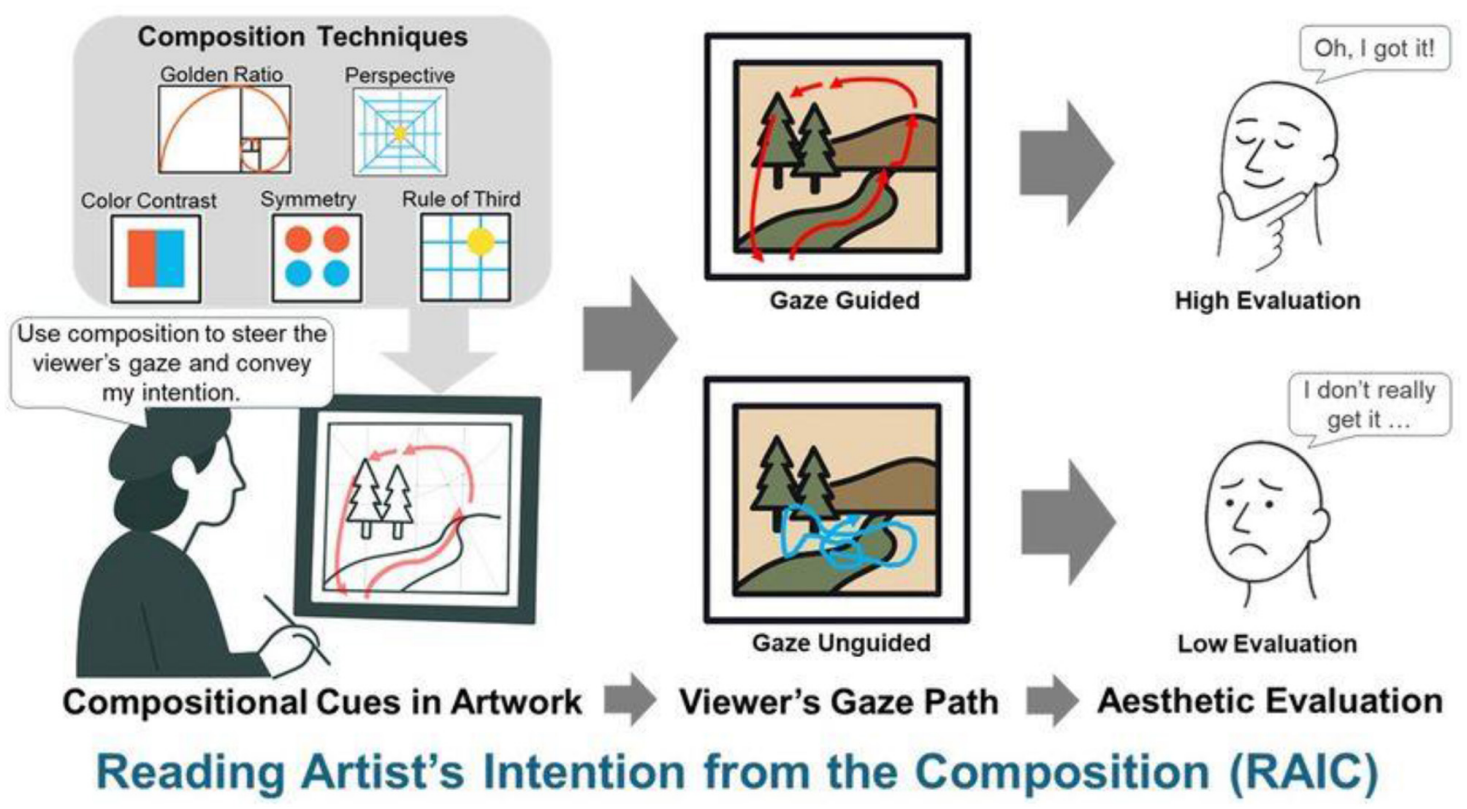
Conceptual framework of Reading an Artist's Intention from the Composition (RAIC). Compositional techniques guide the viewer's gaze, which can either align with the artist's intention (gaze guided) or not (gaze unguided), resulting in differences in aesthetic evaluation.

Figure 2 was missing from the published article. Figure 2 and its caption appear below:

**Figure 2:**
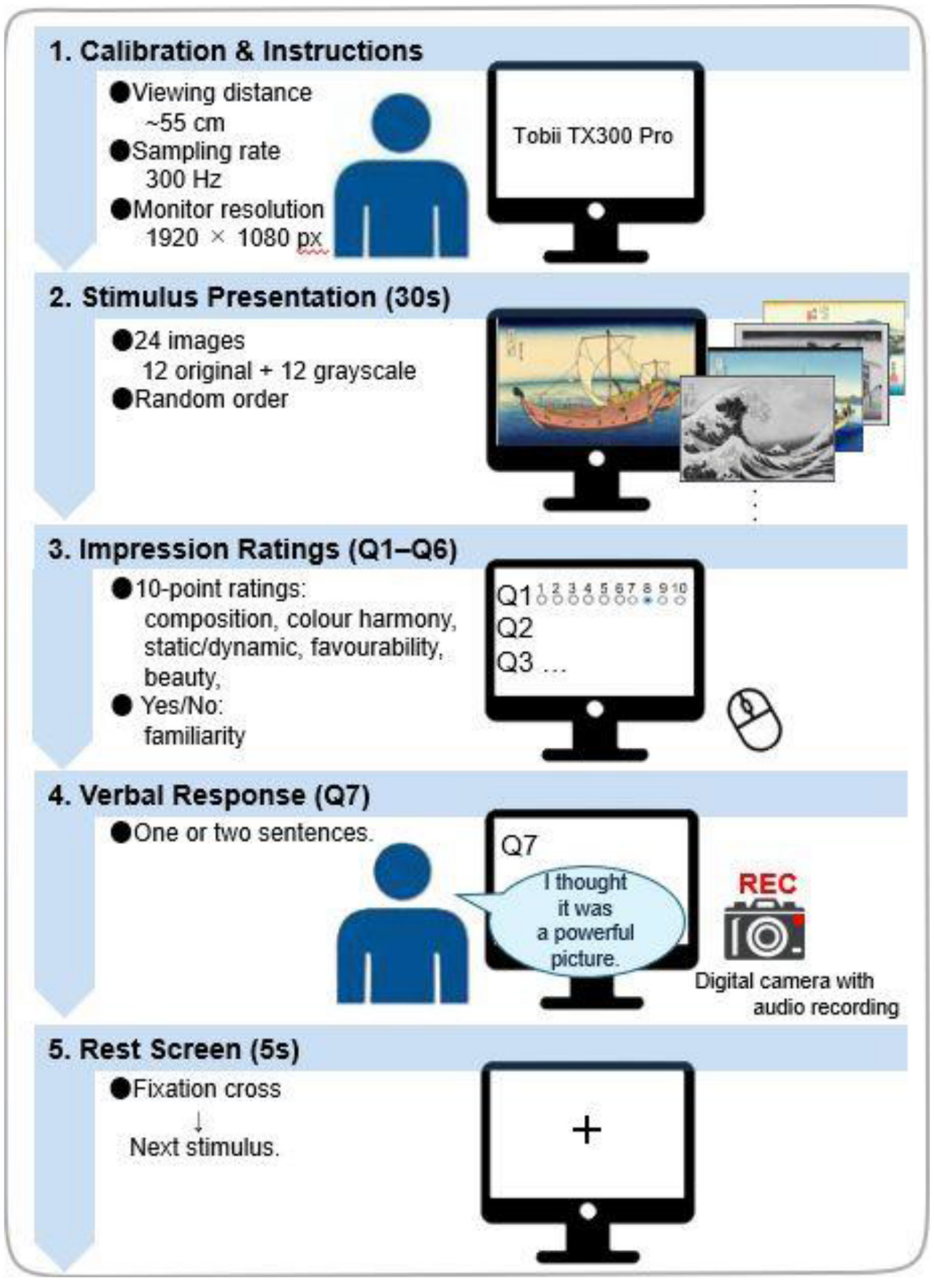
Experimental procedure. Participants sat ~55 cm from the monitor (Tobii TX300 Pro; 300 Hz; 1,920 × 1,080). Twenty-four images (12 original, 12 grayscale) were presented in randomised order for 30 s each while eye movements were recorded. After viewing, participants gave 10-point ratings (composition, colour harmony, static/dynamic, favourability, beauty) and answered in a Yes/No format whether they had seen the painting before, followed by a brief verbal impression (Q7, audio-recorded). Each trial ended with a 5 s fixation cross before the next stimulus.

The original Figure 3 was erroneously labelled as Figure 2. The correct Figure 3 and its caption appear below:

**Figure 3:**
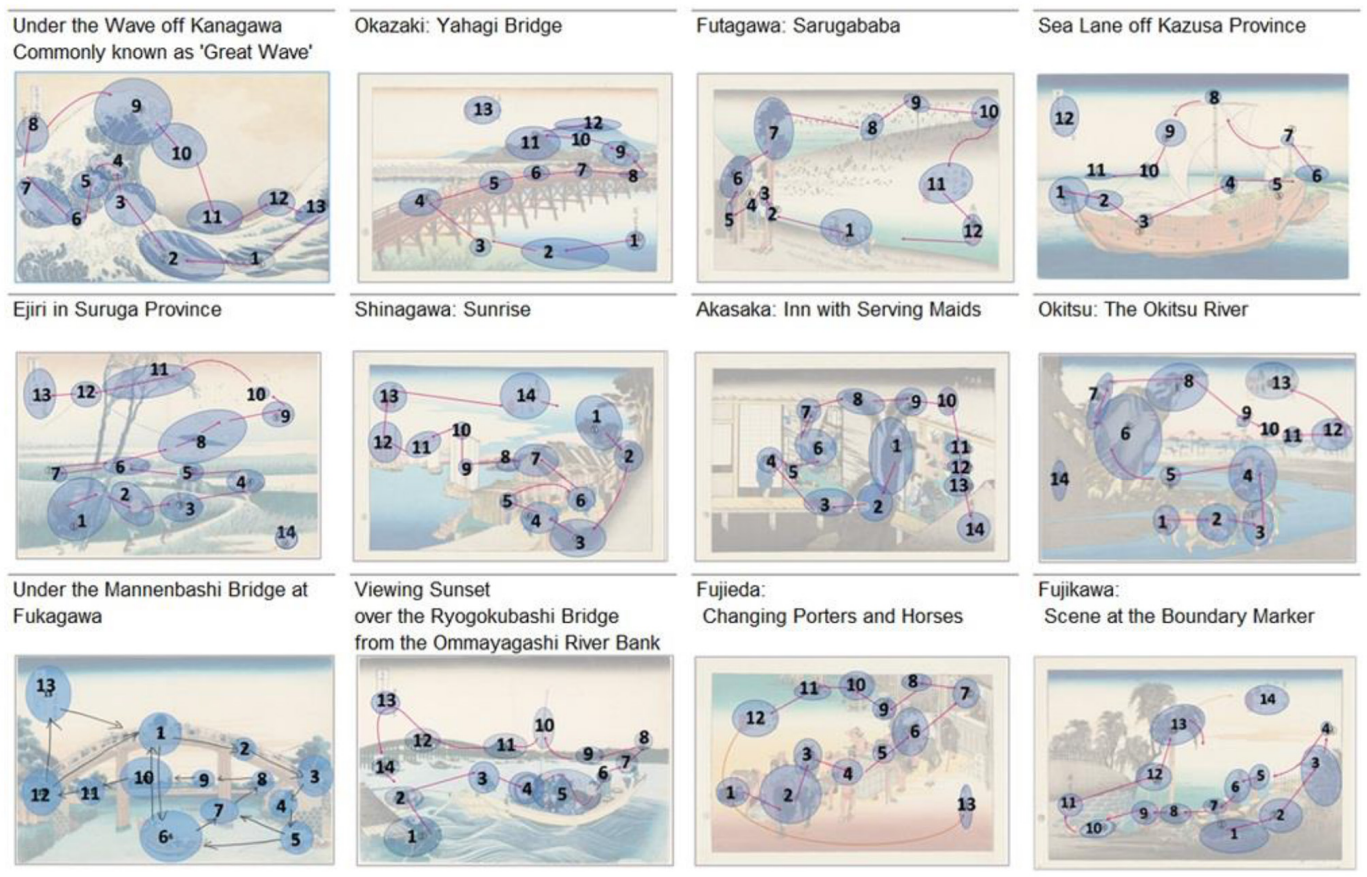
Gaze patterns of Ukiyo-e experts. The patterns for Great Wave, Ejiri, Ryogokubashi, Shinagawa, Okazaki, Kazusa, Akasaka, Okitsu, Fujikawa, Futagawa, and Fujieda were provided by a preparatory school instructor who graduated from the Japanese Painting Conservation and Restoration Laboratory of the Tokyo University of the Arts, whereas those for Mannenbashi were provided by a master's student.

In line with the changes to Figures 2 and 3, the original Figure 3 becomes Figure 4.

The original Figure 4 becomes Figure 5.

The original Figure 5 becomes Figure 6.

The original Figure 6 becomes Figure 7.

The original Figure 7 becomes Figure 8.

The original Figure 8 becomes Figure 9.

The original Figure 9 becomes Figure 10.

A correction has been made to the following sentence in **2.2 Stimuli:**

“Participants viewed each painting for 30 s and rated various aspects such as composition and “beauty” on a 10-point scale; of the 101 paintings, six with particularly high impression ratings for “beauty” and “liking” (mean = 7.85, SD = 0.70) and six with low impression ratings (mean = 4.34, SD = 0.62) were selected for this experiment employed as stimuli (mean = 6.17, SD = 2.17).”

The corrected sentence appears below:

“Participants viewed each painting for 30 s and rated various aspects such as composition and “beauty” on a 10-point scale; of the 101 paintings, six with particularly high impression ratings for “beauty” and “favourability” (mean = 7.85, SD = 0.70) and six with low impression ratings (mean = 4.34, SD = 0.62) were selected for this experiment employed as stimuli (mean = 6.17, SD = 2.17).”

A correction has been made to the following sentence in **2.3 Procedure:**

“The participants viewed the paintings on the PC display sequentially and rated their impressions on a 10-point scale (1 = most negative, 10 = most positive) for the following attributes in the order presented: the “quality of composition,” “integrity of colour harmony,” “static or dynamic,” “liking,” and “beauty” (see Carbon, 2017).”

The corrected sentence appears below:

“The participants viewed the paintings on the PC display sequentially and rated their impressions on a 10-point scale (1 = most negative, 10 = most positive) for the following attributes in the order presented: the “quality of composition,” “integrity of colour harmony,” “static or dynamic,” “favourability,” and “beauty” (see Carbon, 2017).”

A correction has been made to the following sentence in **2.4 Data analysis for gaze pattern:**

“Furthermore, to reflect the relative differences in the evaluation of each painting obtained in the pilot experiment, the normalised impression evaluation values of “beauty” and “liking” were classified by k-means into three groups: high, medium, and low evaluation groups.”

The corrected sentence appears below:

To reflect the relative differences in the evaluation of each painting obtained in the pilot experiment, the normalised impression evaluation values of “beauty” and “favourability” were classified by k-means into three groups: high, medium, and low evaluation groups.

A correction has been made to the following sentence and figure citation in **2.5 Gaze patterns of** ***Ukiyo-e*** **experts:**

“As shown in Figure 1, Kazusa contained 12 ROIs; Great Wave, Mannenbashi, Okazaki, Futagawa, and Fujieda contained 13 ROIs; and the remaining works contained 14.

The correct sentence appears below:

“As shown in Figure 3, Kazusa and Futagawa contained 12 ROIs, Great Wave, Mannenbashi, Okazaki, and Fujieda contained 13 ROIs; and the remaining works contained 14.

A correction has been made to the figure citation in the following sentence in **2.6 EMHMM-based modelling and VH-LL/CVB metrics (gaze–rating link):**

“As an illustration, Figure 3 presents the transition probabilities for Shinagawa over the first 12 s.”

The corrected sentence appears below:

“As an illustration, Figure 4 presents the transition probabilities for Shinagawa over the first 12 s.”

A correction has been made to the following sentence and figure citations in **2.6 EMHMM-based modelling and VH-LL/CVB metrics (gaze–rating link):**

“For each image, the likelihood based on gaze-transition probabilities (VH-LL) and the CVB derived from normalised impression ratings of “beauty” and “favorability” were calculated. The results are presented in Figures 3–6.”

The corrected sentence and figure citations appear below:

“For each image, the likelihood based on gaze-transition probabilities (VH-LL) and the CVB derived from normalised impression ratings of “beauty” and “favourability” were calculated. The results are presented in Figures 5–8.”

A correction has been made to the following figure citations in the bullet points in **2.6 EMHMM-based modelling and VH-LL/CVB metrics (gaze–rating link):**

- “6 highly-rated (Great Wave, Shinagawa, Okazaki, Mannenbashi, Ejiri, Ryogokubashi): CVB in Figure 4 > CVB in Figure 5 → Consistent with the hypothesis.”
- “6 low-rated (Futagawa, Kazusa, Okitsu, Fujikawa, Fujieda, Akasaka): CVB in Figure 4 < CVB in Figure 5 → Consistent with the hypothesis.”
- “5 highly-rated (Great Wave, Mannenbashi, Ejiri, Futagawa, Shinagawa): CVB in Figure 6 > CVB in Figure 7 → Consistent with the hypothesis.”
- “7 low-rated (Okazaki, Ryogokubashi, Kazusa, Okitsu, Fujikawa, Fujieda, Akasaka): CVB in Figure 6 < CVB in Figure 7 → Consistent with the hypothesis.”

The corrected figure citations appear below:

- “6 highly-rated (Great Wave, Shinagawa, Okazaki, Mannenbashi, Ejiri, Ryogokubashi): CVB in Figure 5 > CVB in Figure 6 → Consistent with the hypothesis.”
- “6 low-rated (Futagawa, Kazusa, Okitsu, Fujikawa, Fujieda, Akasaka): CVB in Figure 5 < CVB in Figure 6 → Consistent with the hypothesis.”
- “5 highly-rated (Great Wave, Mannenbashi, Ejiri, Futagawa, Shinagawa): CVB in Figure 7 > CVB in Figure 8 → Consistent with the hypothesis.”
- “7 low-rated (Okazaki, Ryogokubashi, Kazusa, Okitsu, Fujikawa, Fujieda, Akasaka): CVB in Figure 7 < CVB in Figure 8 → Consistent with the hypothesis.”

A correction has been made to the following sentence in **2.6 EMHMM-based modelling and VH-LL/CVB metrics (gaze–rating link):**

“Case for “liking” (Table 6)”

The corrected sentence appears below:

“Case for “favourability” (Table 6)

A correction has been made to the following sentence in **3 Results:**

“Across images, beauty showed stronger and earlier RAIC effects than liking, with the clearest divergences within the first 6 s (notably Great Wave, Ejiri, Ryogokubashi, Futagawa, Okitsu, Fujikawa, Akasaka).”

The corrected sentence appears below:

“Across images, beauty showed stronger and earlier RAIC effects than favourability, with the clearest divergences within the first 6 s (notably Great Wave, Ejiri, Ryogokubashi, Futagawa, Okitsu, Fujikawa, and Akasaka).

A correction has been made to the following sentence in **3.1 CVB summary:**

“Table 6 presents the results of statistical tests for significant differences in CVB related to “liking” between the high and low evaluation groups, using the same procedure as that applied for “beauty.””

The corrected sentence appears below:

“Table 6 presents the results of statistical tests for significant differences in CVB related to “favourability” between the high and low evaluation groups, using the same procedure as that applied for “beauty.””

A correction has been made to the caption of **Table 3**, the published version appears below:

“Number of participants per group by the impressions, per image; “liking.””

The correct caption appears below:

“Number of participants per group by the impressions, per image; “Favourability.””

A correction has been made to the caption of **Table 6**, the published version appears below:

“Normalised “liking” and correlation of VH-LL and “liking.””

The correct caption appears below:

“Normalised “Favourability” and correlation of VH-LL and “Favourability.””

A correction has been made to the caption of **Figure 7 (previously Figure 6)**. The published caption appears below:

“CVB for “favorability” (participants in the high-evaluation group). Solid lines represent the focused model **(a)**, and dashed lines represent the explorative model **(b)**. Each point represents the mean CVB of participants who rated the 12 paintings higher for “favorability” (see Table 3), calculated at 3, 6, 9, 15, and 30 s. The high-evaluation group (Great Wave, Mannenbashi, Ejiri, Futagawa, and Shinagawa) generally showed higher CVB under the focused model, consistent with the hypothesis.”

The corrected caption appears below:

CVB for “favourability” (participants in the high-evaluation group). Solid lines represent the focused model **(a)**, and dashed lines represent the explorative model **(b)**. Each point represents the mean CVB of participants who rated the 12 paintings higher for “favourability” (see Table 3), calculated at 3, 6, 9, 15, and 30 s.

A correction has been made to the caption **of Figure 8 (previously Figure 7)**. The published caption appears below:

“CVB for “favorability” (participants in the low-evaluation group). Solid lines represent the focused model **(a)**, and dashed lines represent the explorative model **(b)**. Each point represents the mean CVB of participants who rated the 12 paintings lower for “favorability” (see Table 3), calculated at 3, 6, 9, 15, and 30 s. In contrast, the low-evaluation group (Akasaka, Okazaki, Ryogokubashi, Kazusa, Okitsu, Fujikawa, and Fujieda) tended to show stronger CVB under the explorative model, indicating weaker correspondence with focused expert strategies.”

The corrected caption appears below:

“CVB for “favourability” (participants in the low-evaluation group). Solid lines represent the focused model **(a)**, and dashed lines represent the explorative model **(b)**. Each point represents the mean CVB of participants who rated the 12 paintings lower for “favourability” (see Table 3), calculated at 3, 6, 9, 15, and 30 s. In contrast, the low-evaluation group (Akasaka, Okazaki, Ryogokubashi, Kazusa, Okitsu, Fujikawa, and Fujieda) tended to show stronger CVB under the explorative model, indicating weaker correspondence with focused expert strategies.”

A correction has been made to the figure citations in the following sentence in **3.2 Processing fluency measured by pupil size:**

“Figures 8, 9 and Table 7 show the results of the pupil size analysis for the high- and low-evaluation groups, classified via k-means clustering based on normalised beauty ratings.”

The corrected figure citations appear below:

“Figures 9, 10 and Table 7 show the results of the pupil size analysis for the high- and low-evaluation groups, classified via k-means clustering based on normalised beauty ratings.”

A correction has been made in the **Acknowledgments** statement for clarity. The published statement can be seen below:

“The research was conducted at the Graduate School of Arts and Sciences, The University of Tokyo. The author also acknowledges completing doctoral coursework at A University in March 2024 without obtaining a degree.”

The correct statement appears below:

“The research was conducted at the Graduate School of Arts and Sciences, the University of Tokyo. The author also acknowledges completing doctoral coursework at the University in March 2024 without obtaining a degree.”

The original version of this article has been updated.

## Generative AI statement

Any alternative text (alt text) provided alongside figures in this article has been generated by Frontiers with the support of artificial intelligence and reasonable efforts have been made to ensure accuracy, including review by the authors wherever possible. If you identify any issues, please contact us.

