# Supplementary figures and images for: Reading an Artist’s Intention from the Composition (RAIC): eye movements and aesthetic experience in Japanese woodblock prints
Source: Front Psychol. 2025 Nov 12;16:1644803. doi: 10.3389/fpsyg.2025.1644803 (PMC12648216; doi:10.3389/fpsyg.2025.1644803)

Supplementary Material 3

**All the candidate EGP**

| 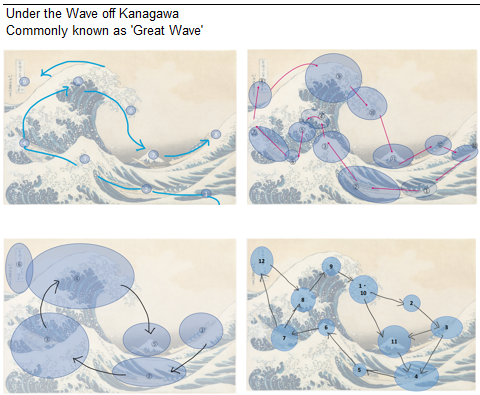 |
| --- |
| 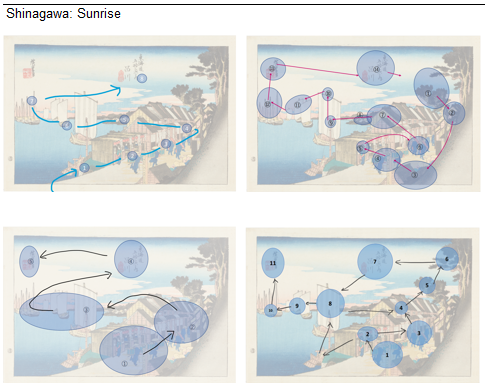 |
| 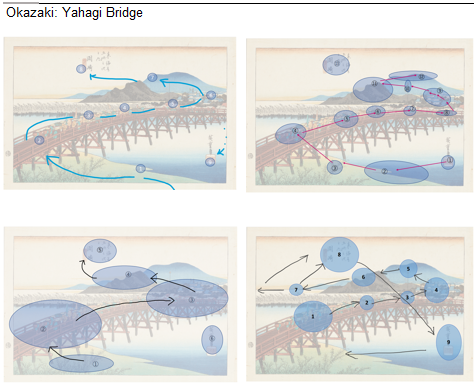 |
| 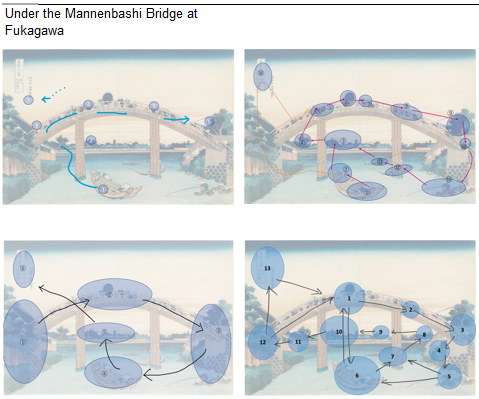 |
| 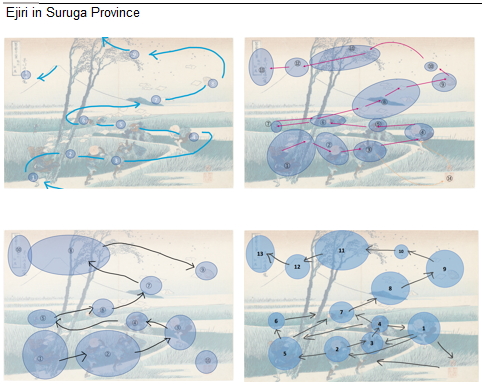 |
| 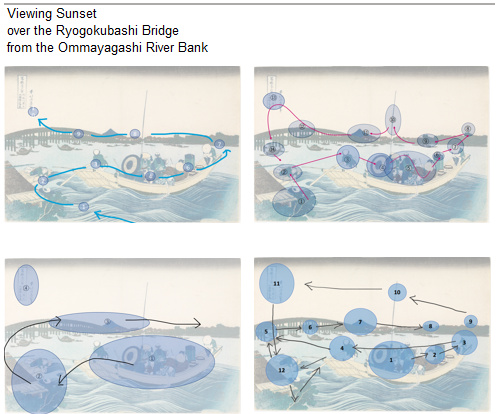 |
| 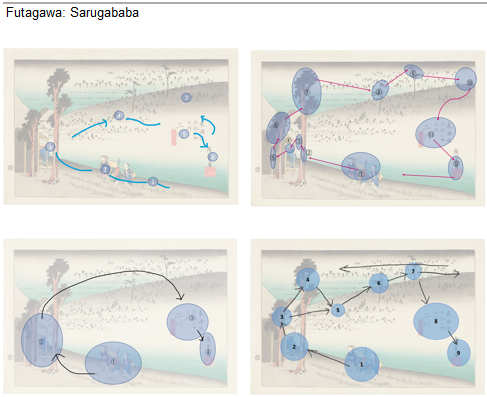 |
| 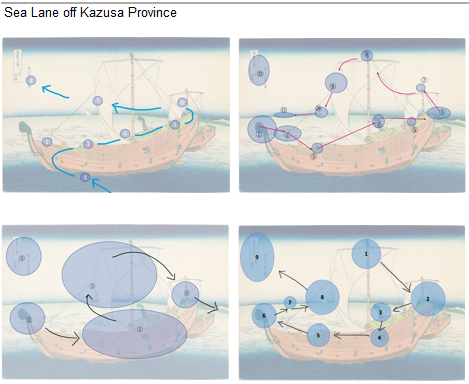 |
| 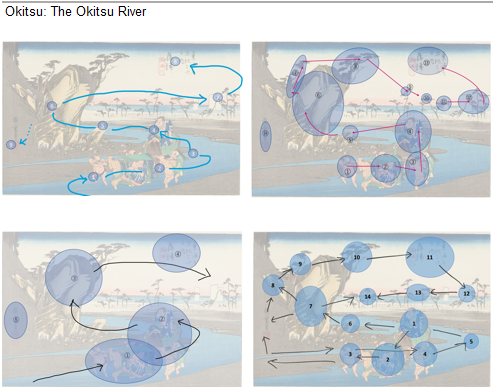 |
| 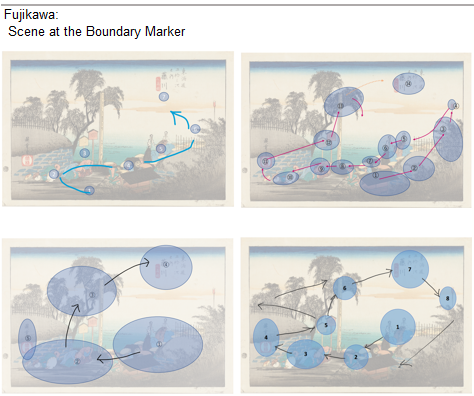 |
| 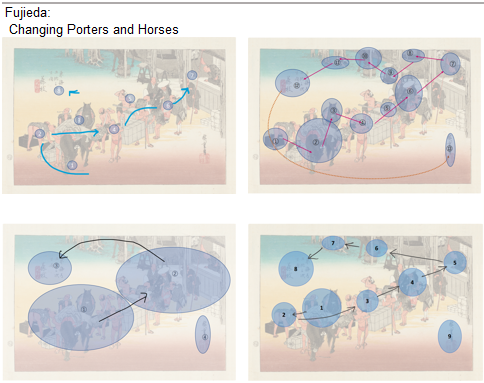 |
| 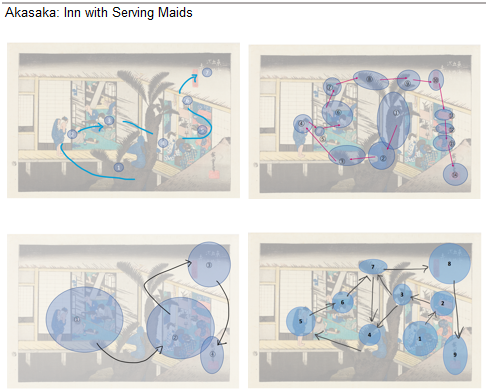 |

Supplement: Supplementary file 1 [file Table_3.docx]
